# Supplementary material for: The Unfolded Protein Response Is a Major Driver of LCN2 Expression in BCR–ABL- and JAK2V617F-Positive MPN
Source: Cancers (Basel). 2021 Aug 21;13(16):4210. doi: 10.3390/cancers13164210 (PMC8391615; doi:10.3390/cancers13164210)
Supplement: Supplementary file 1 [file cancers-13-04210-s001.zip › cancers-1268224-Data S1.pdf]

Figure 3B

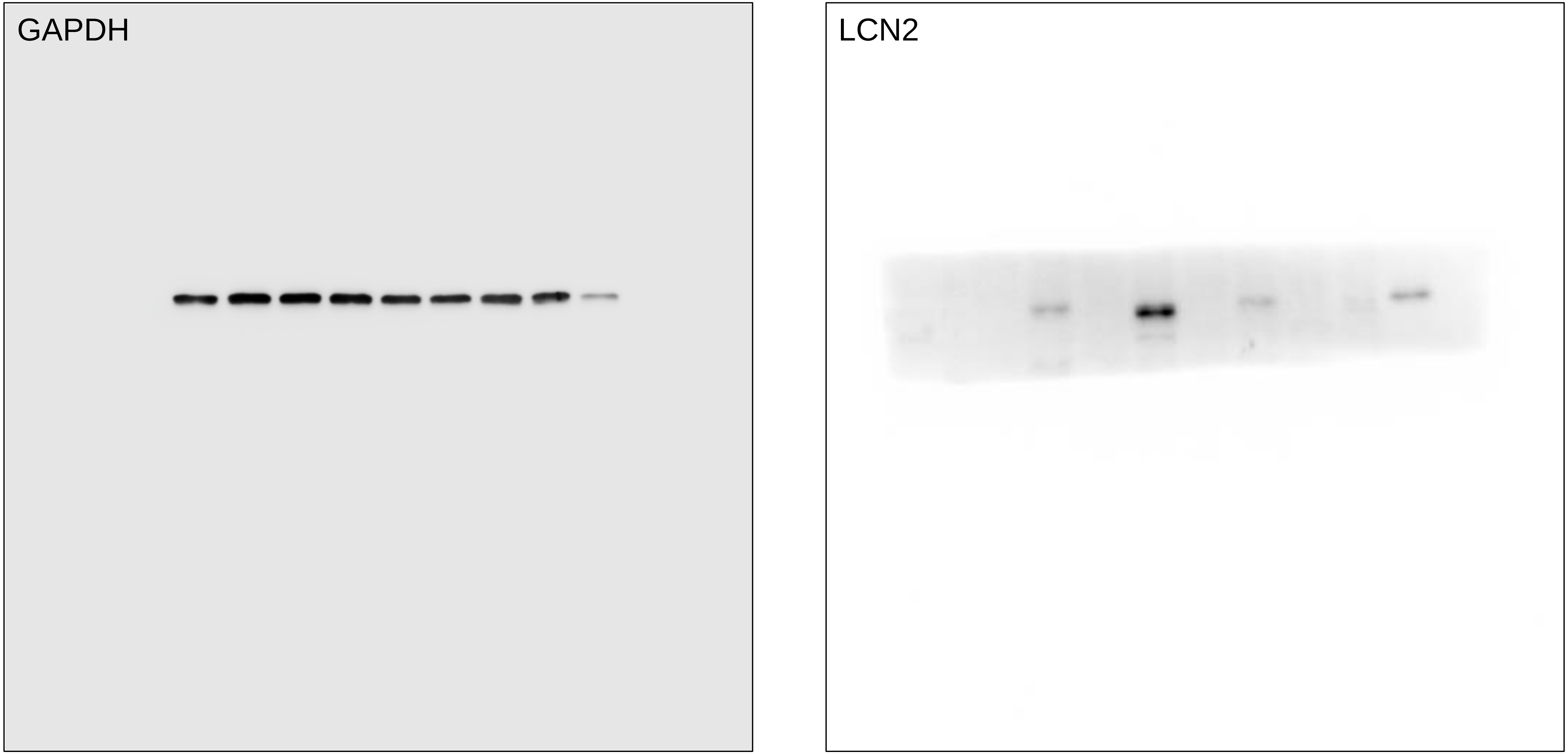

Figure 3B

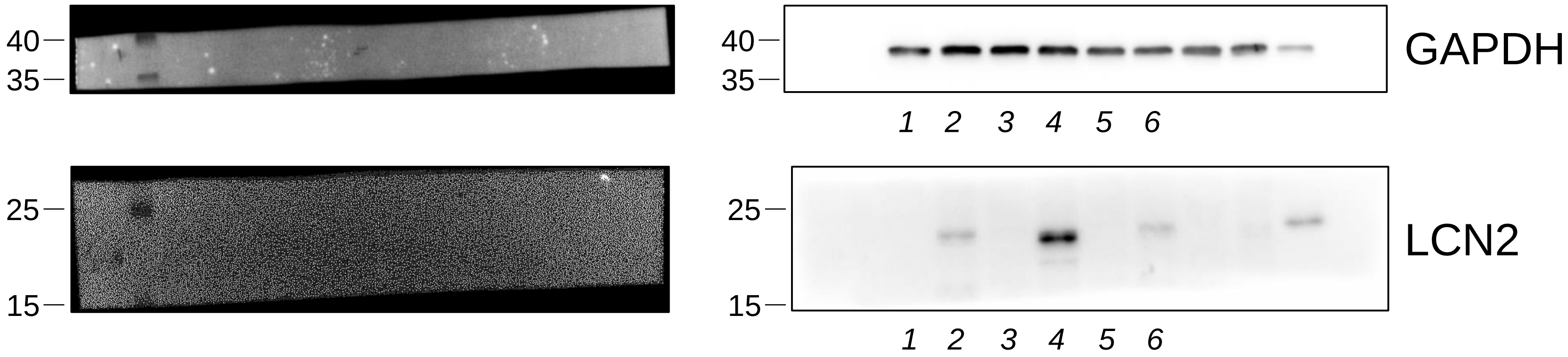

Figure 3B

GAPDH

Lane	optical density
1	1.997.941
2	2.791.770
3	2.775.477
4	2.491.062
5	1.797.234
6	1.651.820

LCN2

Lane	optical density
1	21.950
2	493.577
3	14.536
4	2.579.305
5	29.950
6	356.042

Figure 3E

Empty Vector

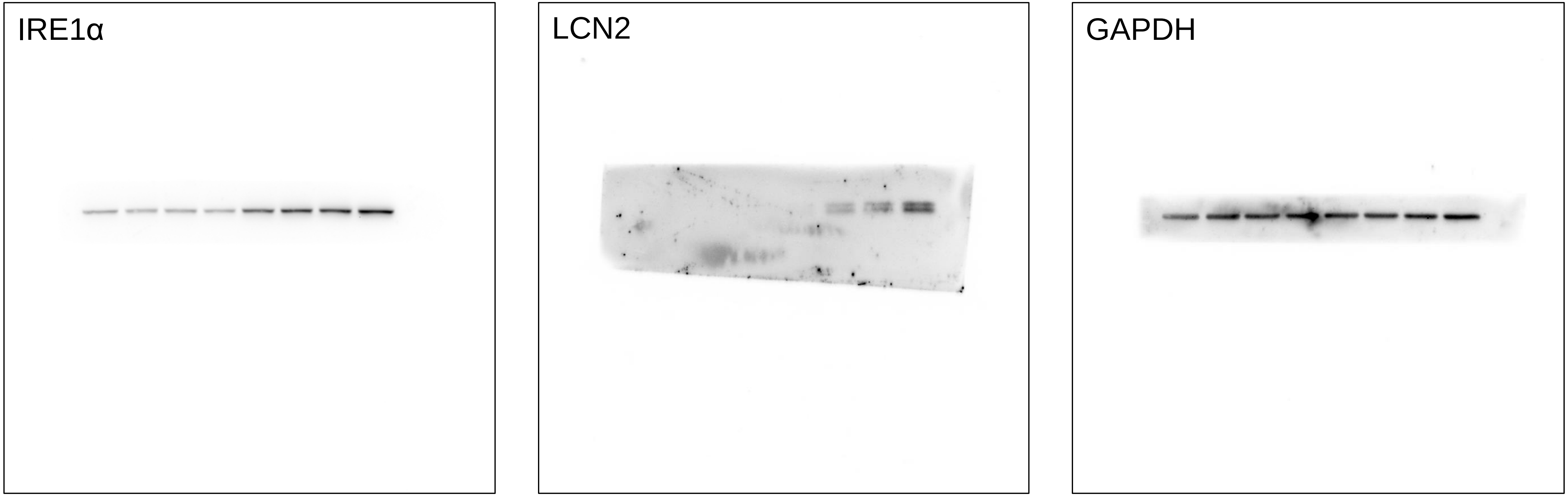

Figure 3E

Empty Vector

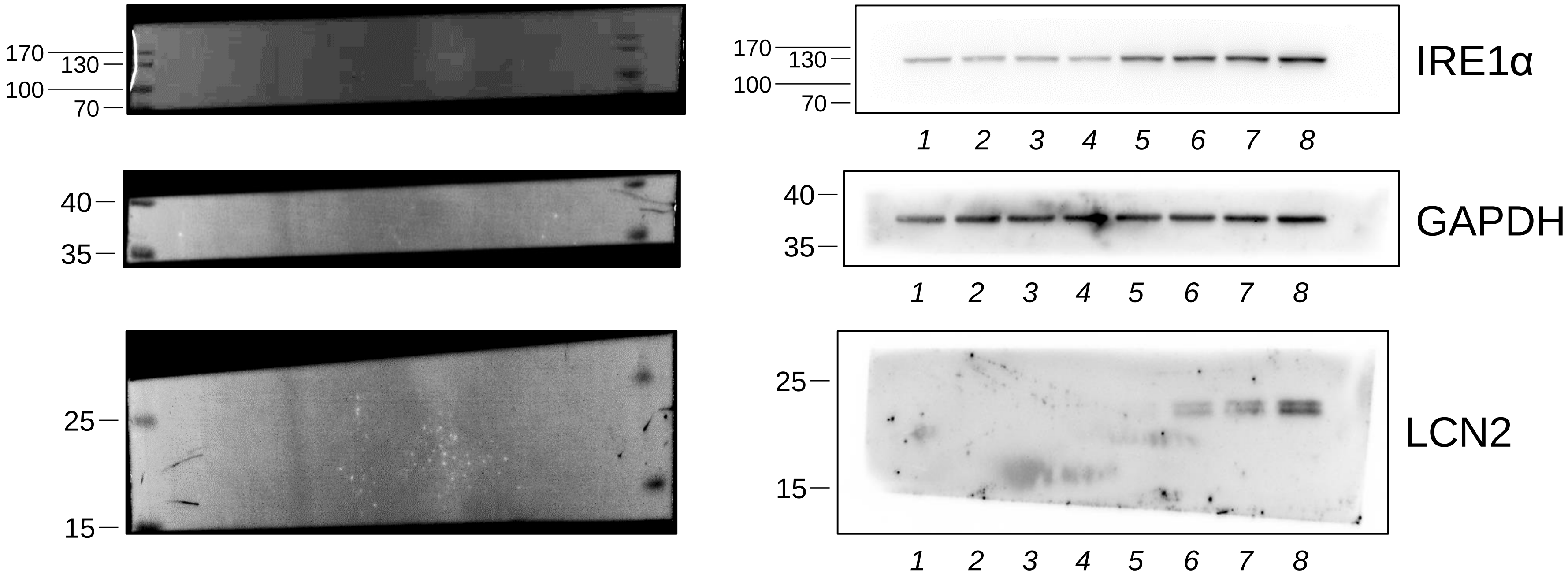

Figure 3E

Empty Vector

IRE1α

Lane	optical density
1	583.335
2	442.920
3	483.627
4	412.799
5	1.018.577
6	1.472.355
7	1.471.234
8	2.079.477

GAPDH

Lane	optical density
1	1.564.770
2	2.372.184
3	2.472.477
4	3.775.598
5	2.278.648
6	1.891.062
7	1.986.234
8	2.413.941

LCN2

Lane	optical density
1	20.950
2	16.950
3	24.364
4	35.364
5	64.778
6	562.163
7	918.527
8	2.108.770

Figure 3E

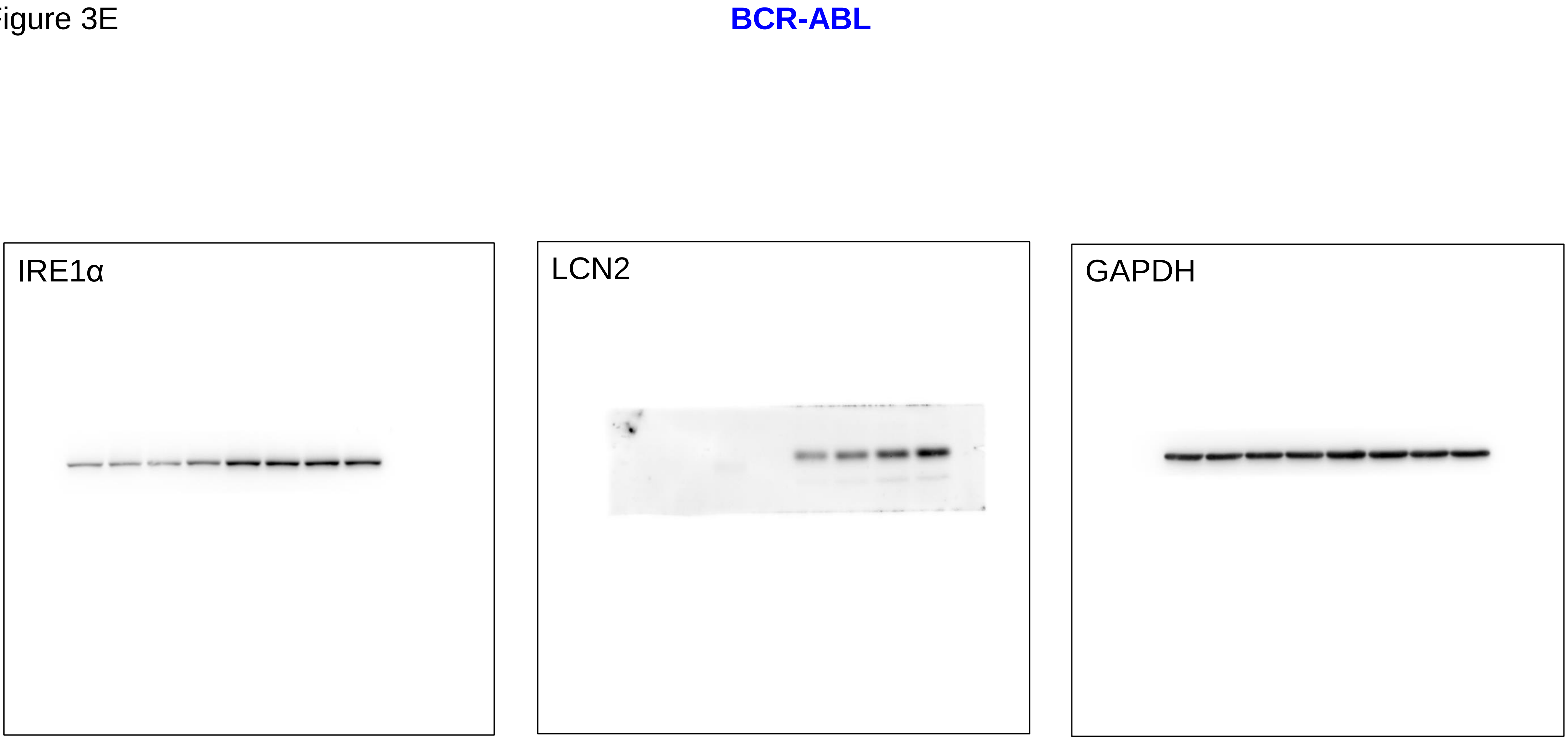

Figure 3E

BCR-ABL

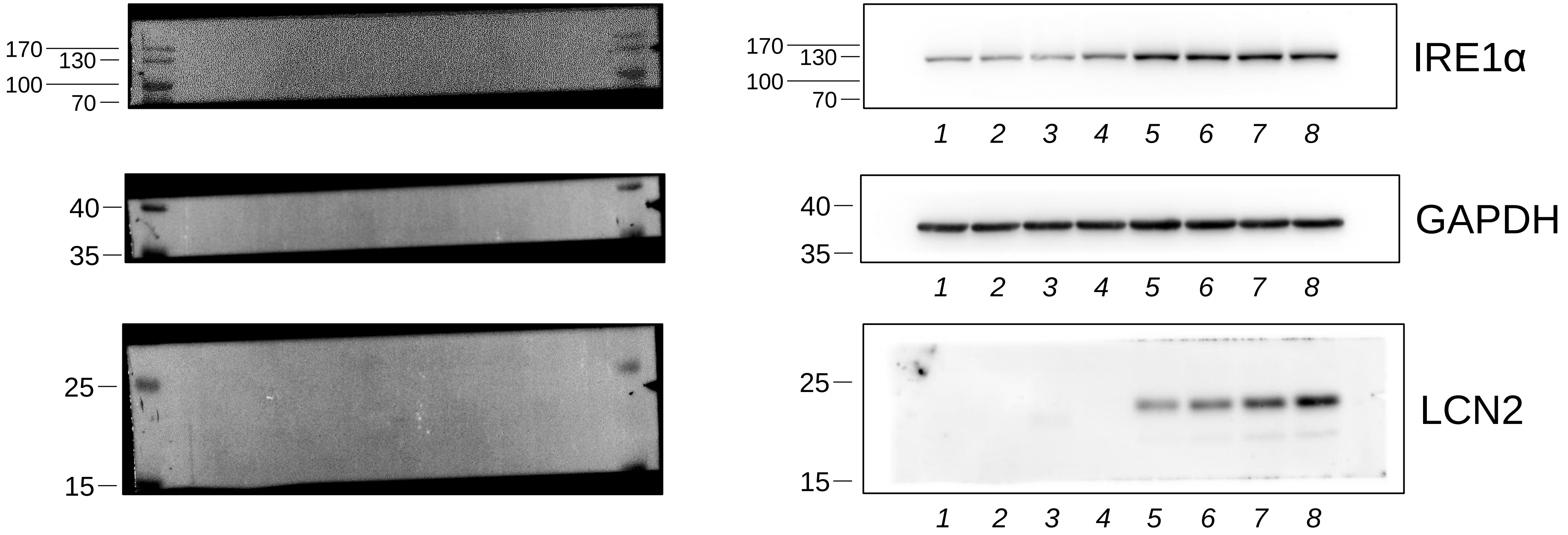

Figure 3E

BCR-ABL

IRE1α

Lane	optical density
1	796.163
2	697.456
3	755.870
4	1.207.234
5	2.430.134
6	2.482.134
7	2.523.134
8	2.403.012

GAPDH

Lane	optical density
1	3.292.376
2	3.431.790
3	3.435.083
4	3.467.083
5	4.238.619
6	3.958.669
7	3.564.134
8	3.428.719

LCN2

Lane	optical density
1	33.657
2	37.728
3	26.364
4	26.657
5	1.408.305
6	1.851.012
7	2.489.426
8	3.095.012

Figure 3E

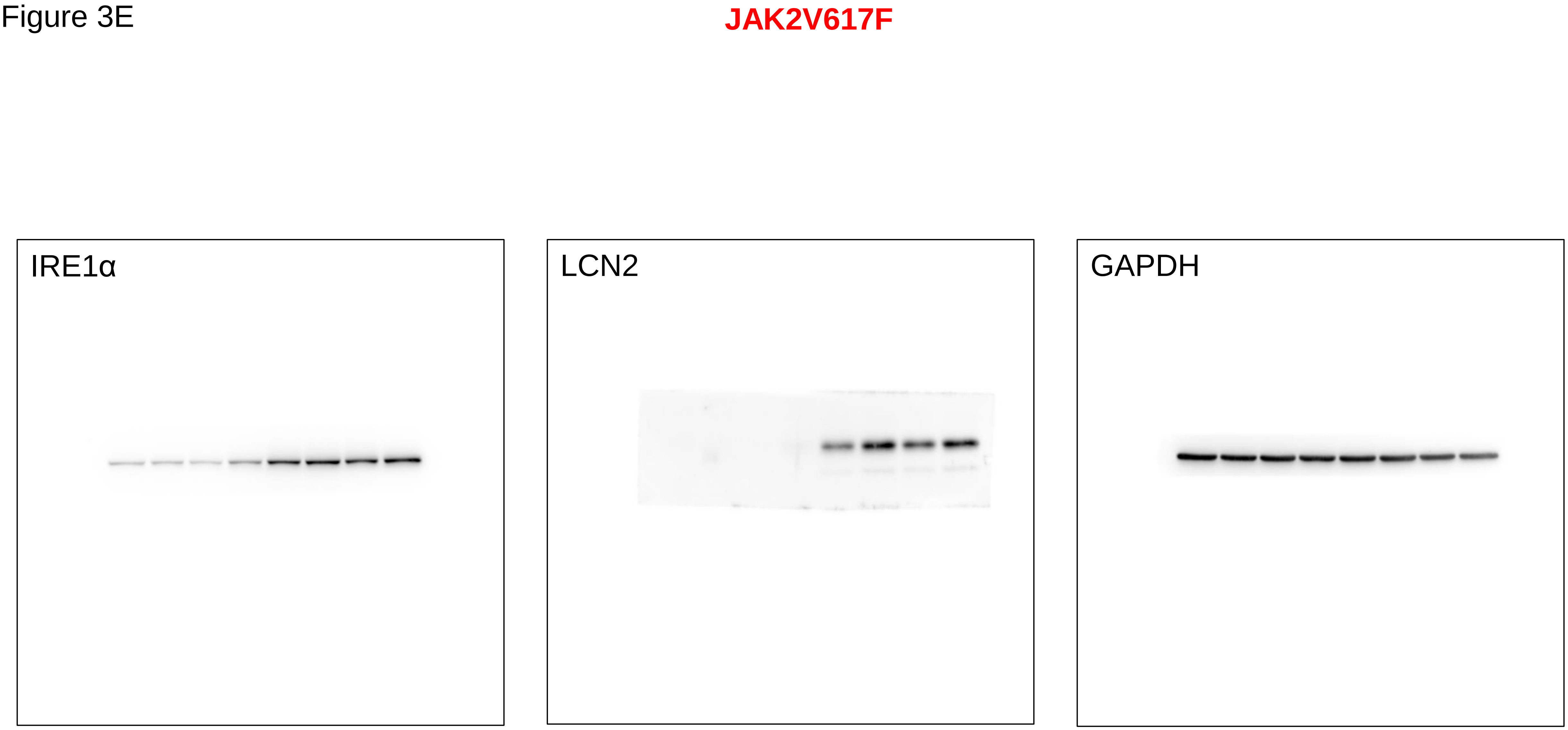

Figure 3E

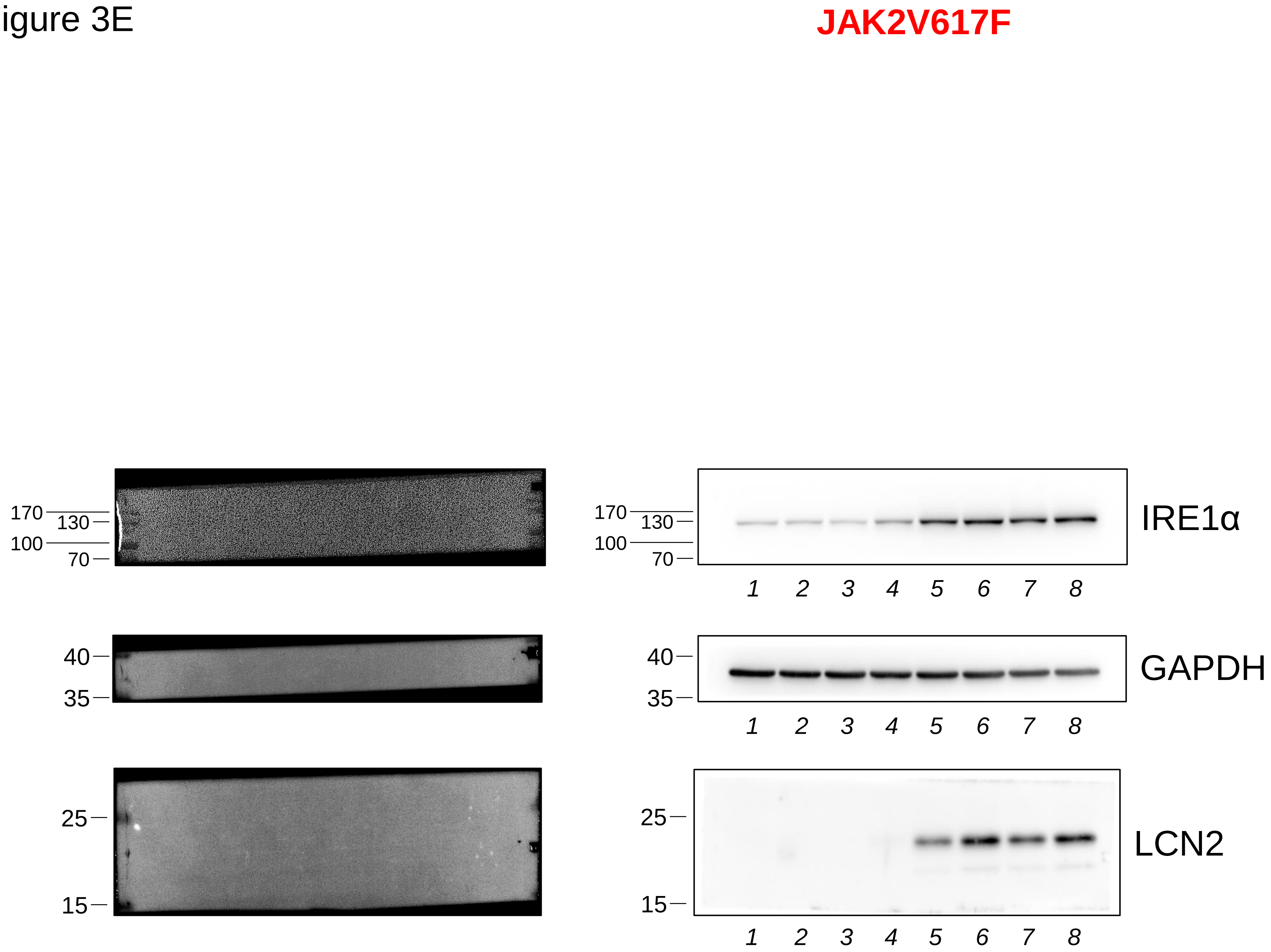
JAK2V617F
170
130
100
70
IRE1α
1 2 3 4 5 6 7 8
40
35
GAPDH
1 2 3 4 5 6 7 8
25
15
LCN2
1 2 3 4 5 6 7 8

Figure 3E

JAK2V617F

IRE1α

Lane	optical density
1	401.799
2	402.627
3	271.678
4	704.870
5	1.804.891
6	2.232.305
7	1.973.012
8	2.505.719

GAPDH

Lane	optical density
1	3.453.669
2	2.917.255
3	2.994.669
4	2.836.548
5	3.014.548
6	2.730.134
7	2.299.891
8	1.964.355

LCN2

Lane	optical density
1	28.778
2	29.950
3	31.657
4	95.950
5	1.748.719
6	3.222.255
7	2.214.598
8	2.954.012

Figure 3G

BCR-ABL

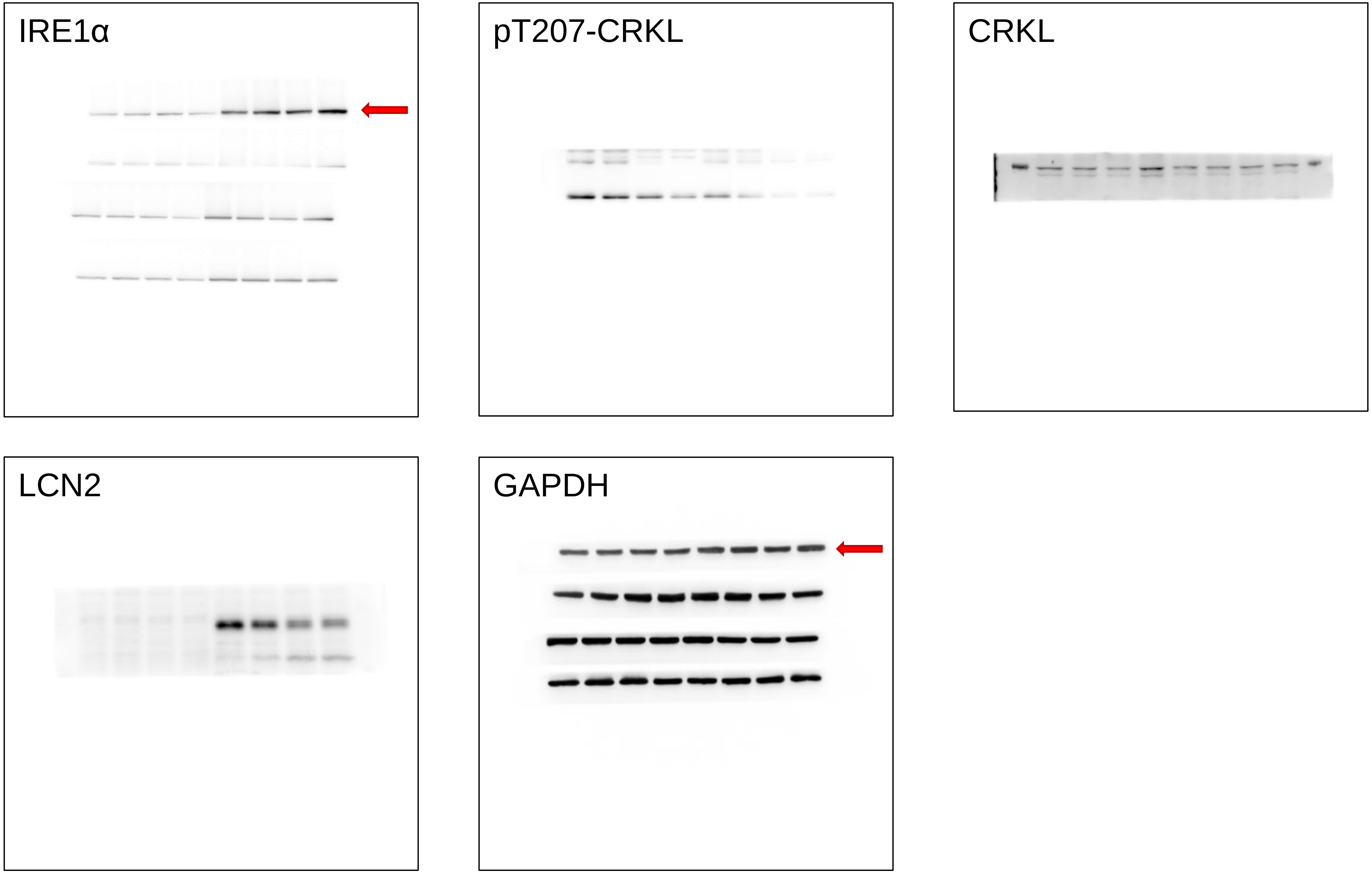

Figure 3G

BCR-ABL

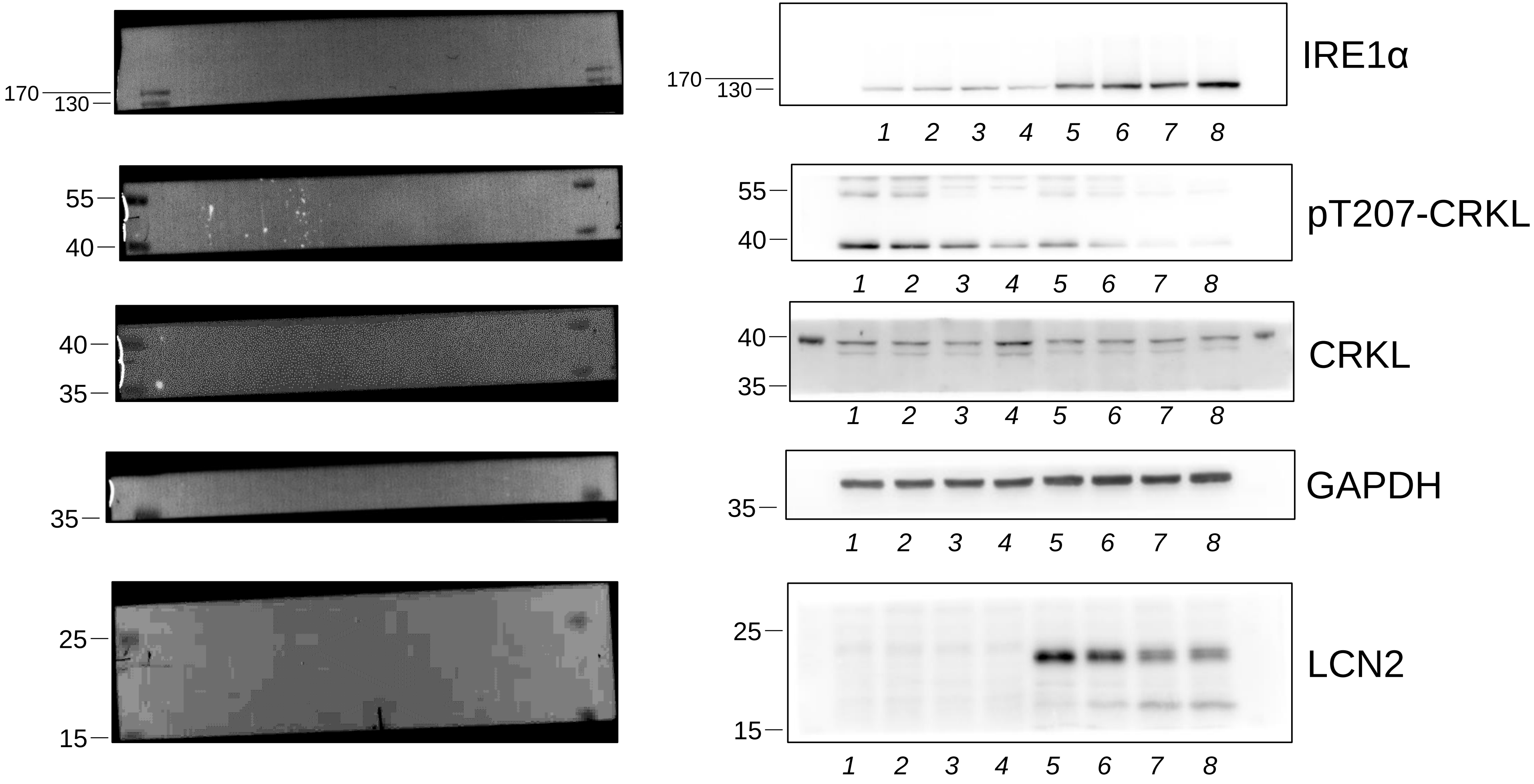

Figure 3G

BCR-ABL

IRE1α

Lane	optical density
1	259.556
2	334.678
3	420.092
4	239.971
5	1.016.991
6	1.496.113
7	1.289.698
8	2.114.820

pT207-CRKL

Lane	optical density
1	1.716.820
2	1.297.870
3	930.870
4	509.920
5	745.456
6	368.920
7	105.192
8	107.607

CRKL

Lane	optical density
1	837.799
2	613.385
3	460.678
4	1.163.042
5	644.042
6	568.920
7	615.456
8	695.991

GAPDH

Lane	optical density
1	3.820.619
2	3.664.619
3	3.930.033
4	4.073.861
5	4.397.569
6	5.460.640
7	4.902.225
8	5.697.347

LCN2

Lane	optical density
1	123.778
2	71.364
3	105.778
4	126.192
5	3.388.619
6	2.581.790
7	1.509.426
8	1.603.841

Figure 3G

JAK2V617F

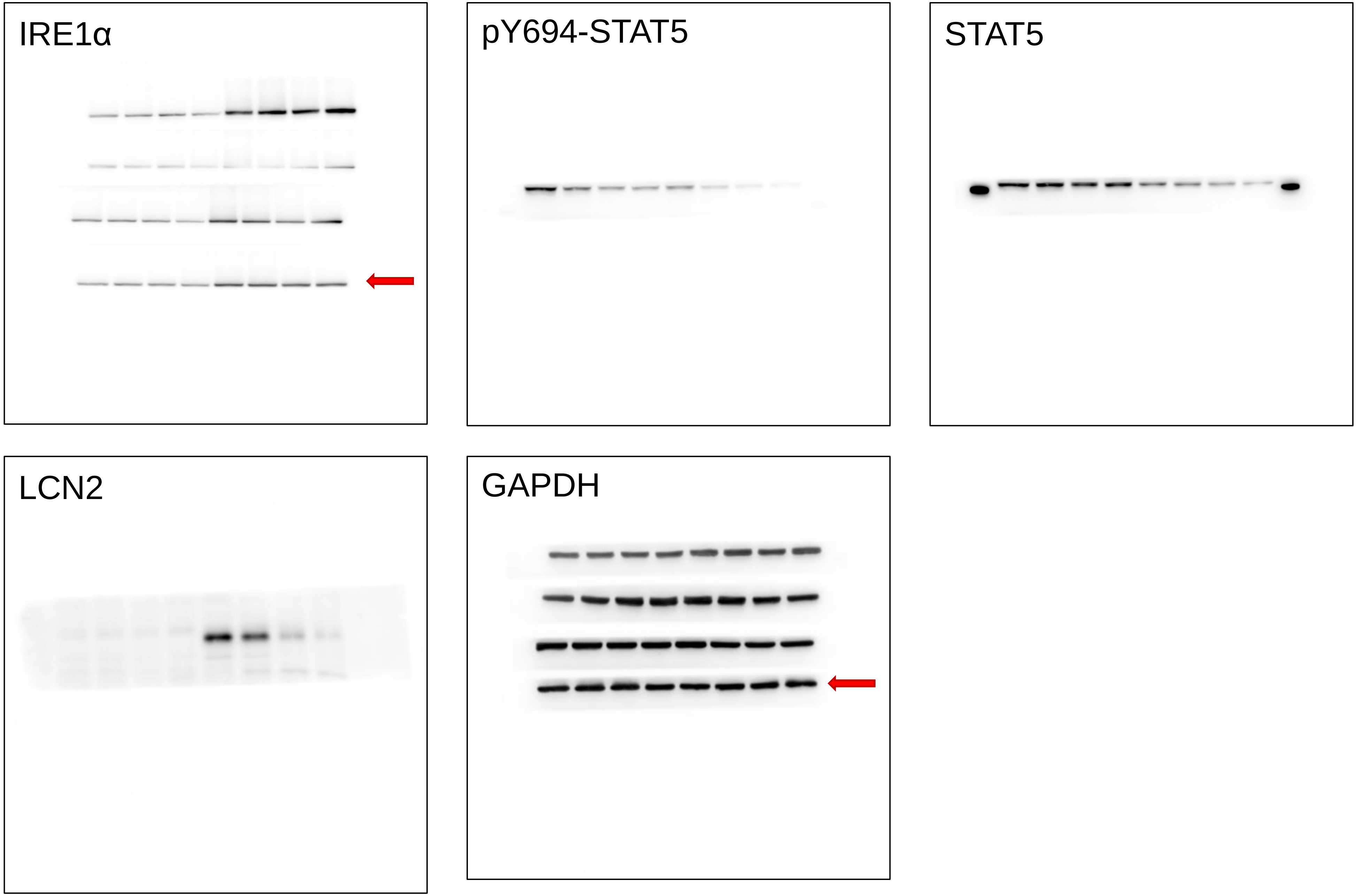

Figure 3G

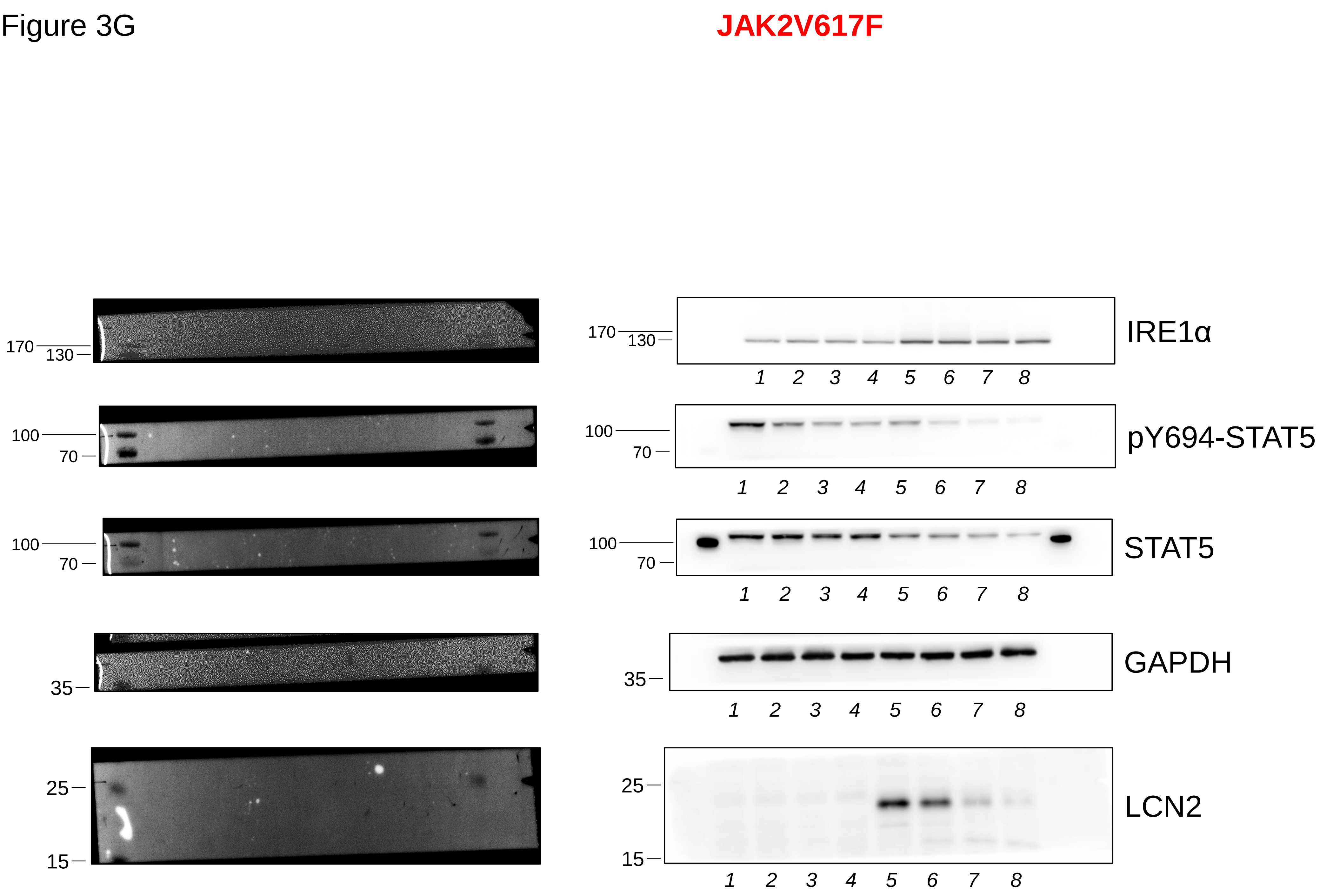
JAK2V617F
170
130
100
70
100
70
35
25
15
170
130
IRE1α
1 2 3 4 5 6 7 8
100
70
pY694-STAT5
1 2 3 4 5 6 7 8
100
70
STAT5
1 2 3 4 5 6 7 8
35
GAPDH
1 2 3 4 5 6 7 8
25
15
LCN2
1 2 3 4 5 6 7 8

Figure 3G

JAK2V617F

IRE1α

Lane	optical density
1	1.145.698
2	1.227.113
3	1.311.355
4	1.131.234
5	2.542.548
6	2.413.719
7	2.355.719
8	2.483.719

pY694-STAT5

Lane	optical density
1	4.876.397
2	2.722.276
3	1.537.962
4	1.380.426
5	1.649.255
6	641.284
7	248.849
8	169.192

STAT5

Lane	optical density
1	6.292.468
2	5.885.296
3	4.546.933
4	4.876.933
5	2.657.326
6	2.301.912
7	1.512.841
8	1.254.841

GAPDH

Lane	optical density
1	7.954.368
2	8.613.953
3	7.924.711
4	7.742.832
5	7.810.125
6	8.209.246
7	8.171.246
8	8.786.489

LCN2

Lane	optical density
1	184.364
2	200.192
3	232.778
4	380.021
5	10.811.459
6	7.670.560
7	1.455.326
8	585.335

Figure 4C

Empty Vector

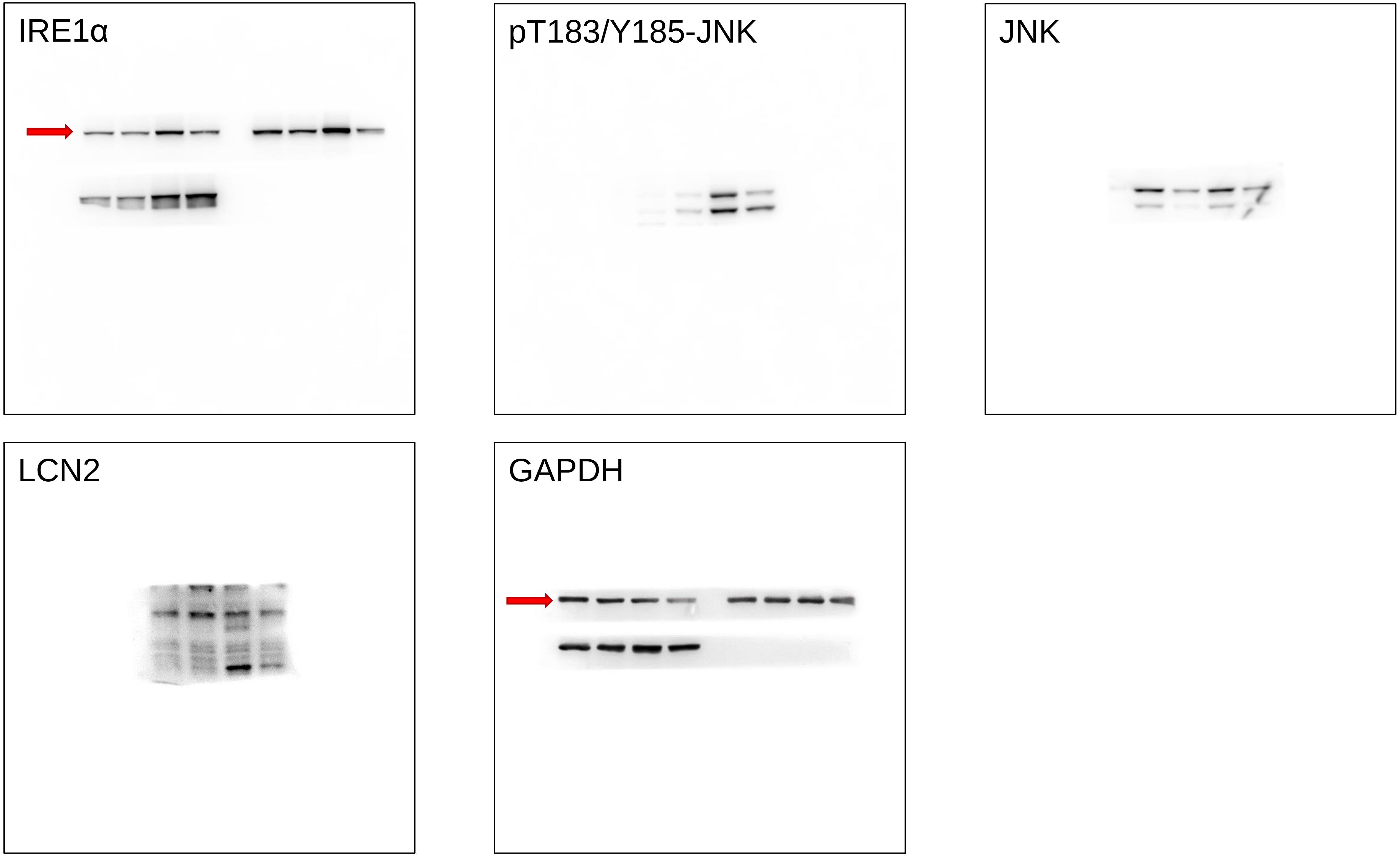

Figure 4C

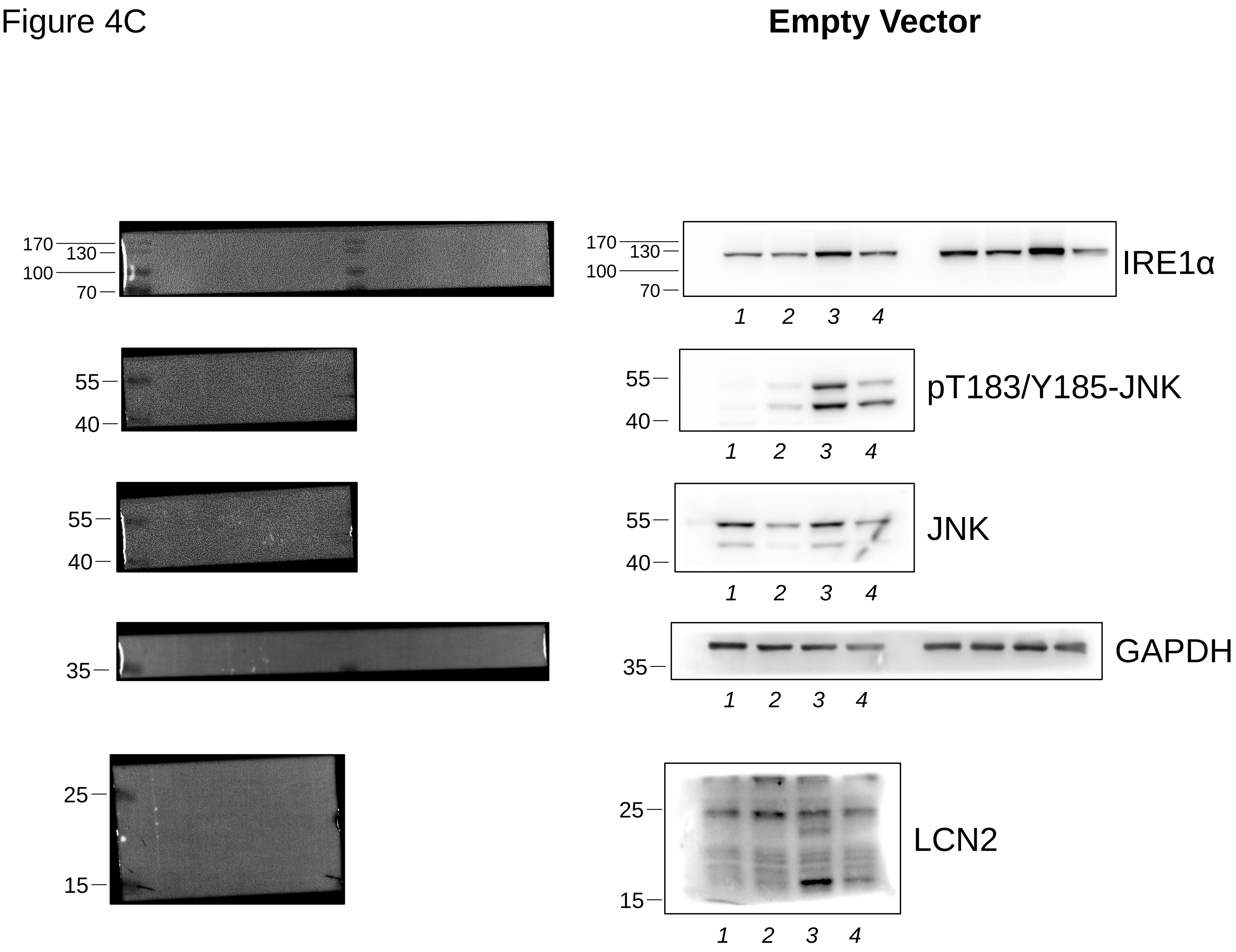
Empty Vector
170
130
100
70
IRE1α
1 2 3 4
55
40
pT183/Y185-JNK
JNK
GAPDH
35
25
15
LCN2

Figure 4C

Empty Vector

IRE1α

Lane	optical density
1	2.253.891
2	2.039.891
3	4.879.276
4	3.040.083

pT183/Y185-JNK

Lane	optical density
1	533.849
2	1.915.326
3	11.544.794
4	6.162.945

JNK

Lane	optical density
1	9.038.773
2	3.917.882
3	9.026.530
4	7.381.711

GAPDH

Lane	optical density
1	4.455.447
2	3.709.276
3	2.992.497
4	2.338.497

LCN2

Lane	optical density
1	43.435
2	27.364
3	675.719
4	54.728

Figure 4C

BCR-ABL

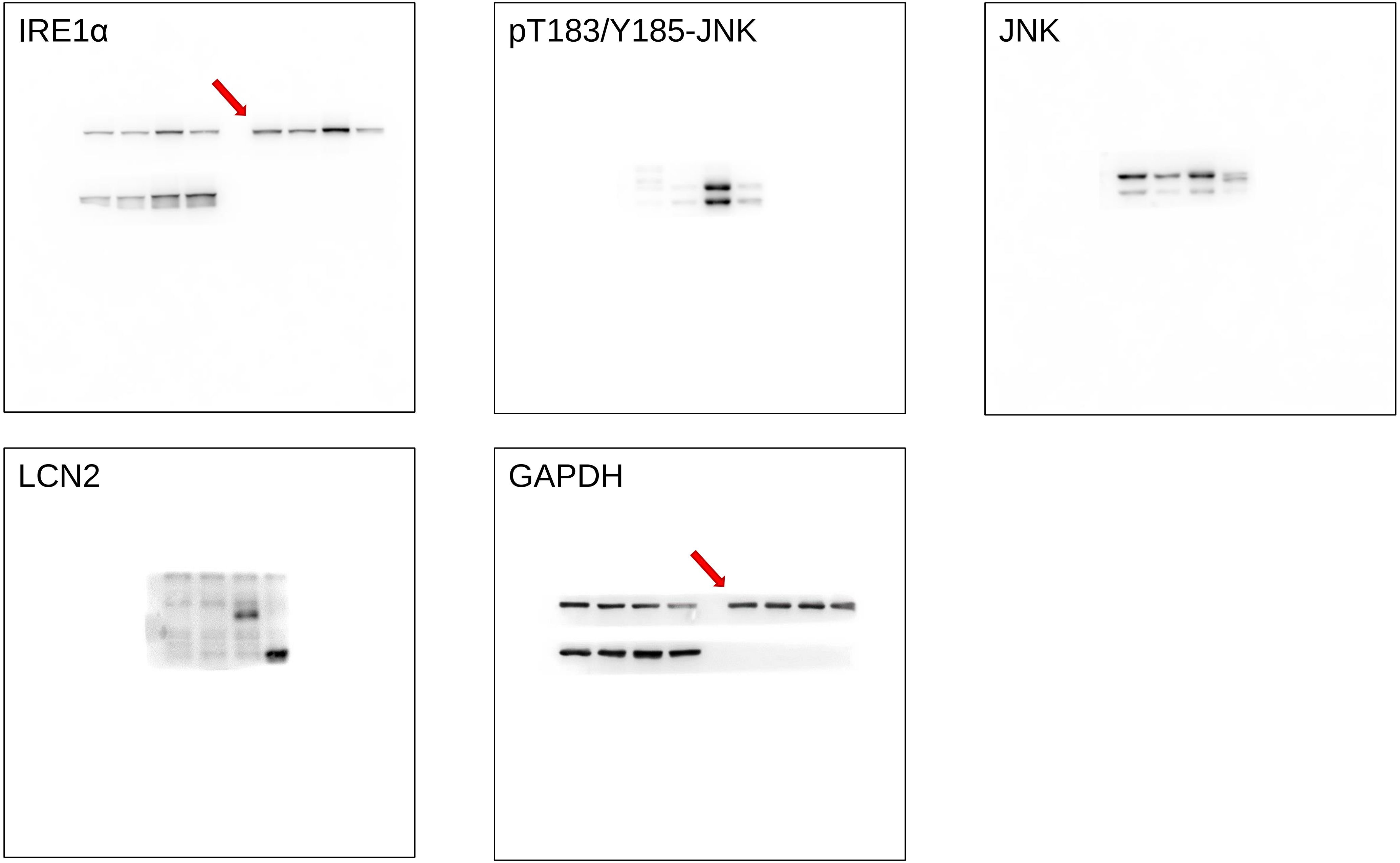

Figure 4C

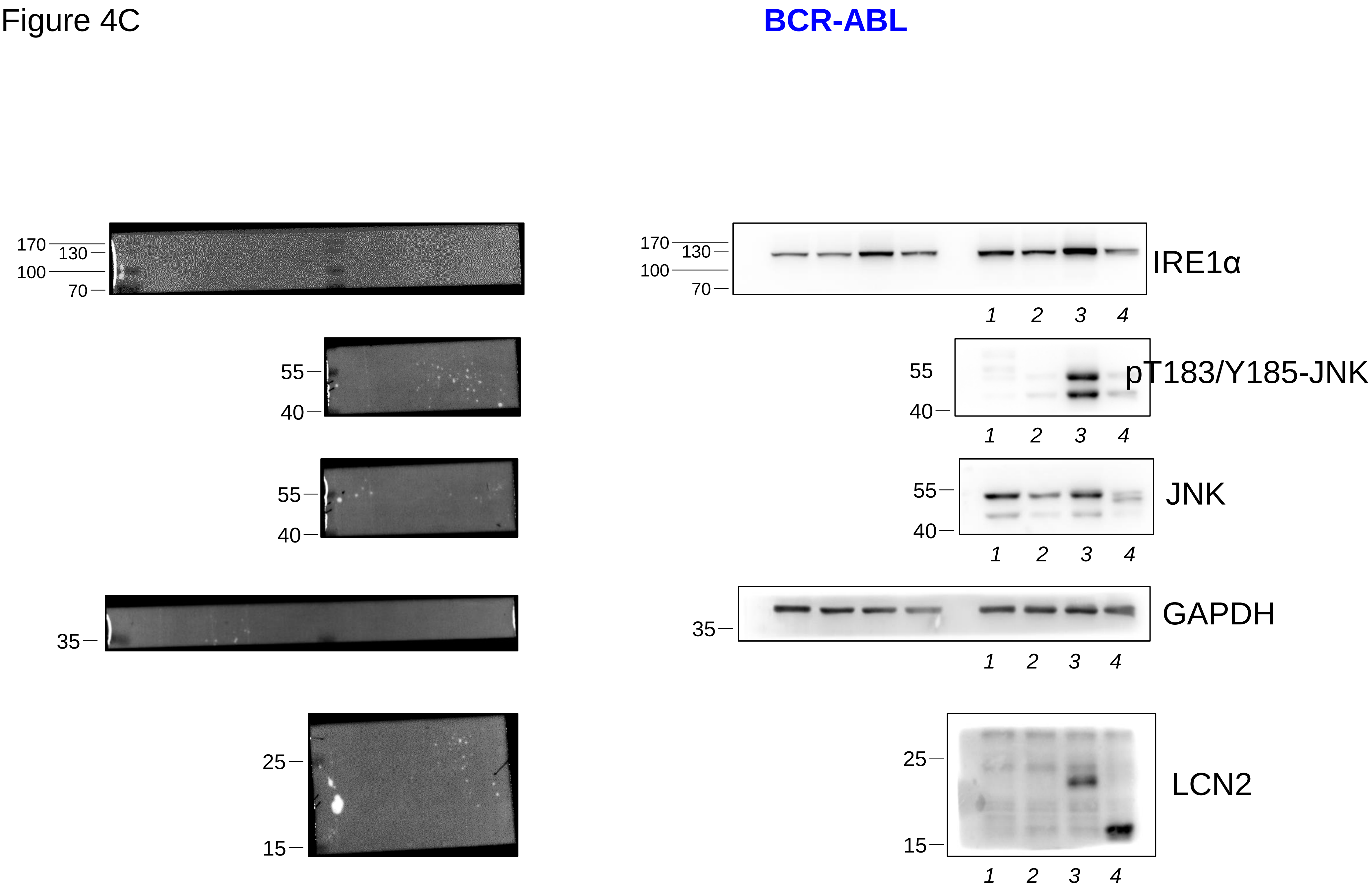
BCR-ABL
170
130
100
70
IRE1α
1 2 3 4
55
40
pT183/Y185-JNK
JNK
35
GAPDH
25
15
LCN2

Figure 4C

BCR-ABL

IRE1α

Lane	optical density
1	6.528.246
2	5.057.589
3	8.531.610
4	3.220.861

pT183/Y185-JNK

Lane	optical density
1	524.435
2	1.225.406
3	15.216.187
4	2.837.640

JNK

Lane	optical density
1	8.015.116
2	4.106.468
3	8.103.945
4	3.479.104

GAPDH

Lane	optical density
1	4.159.811
2	4.799.711
3	5.341.953
4	4.850.418

LCN2

Lane	optical density
1	40.950
2	44.950
3	6.283.560
4	48.950

Figure 4C

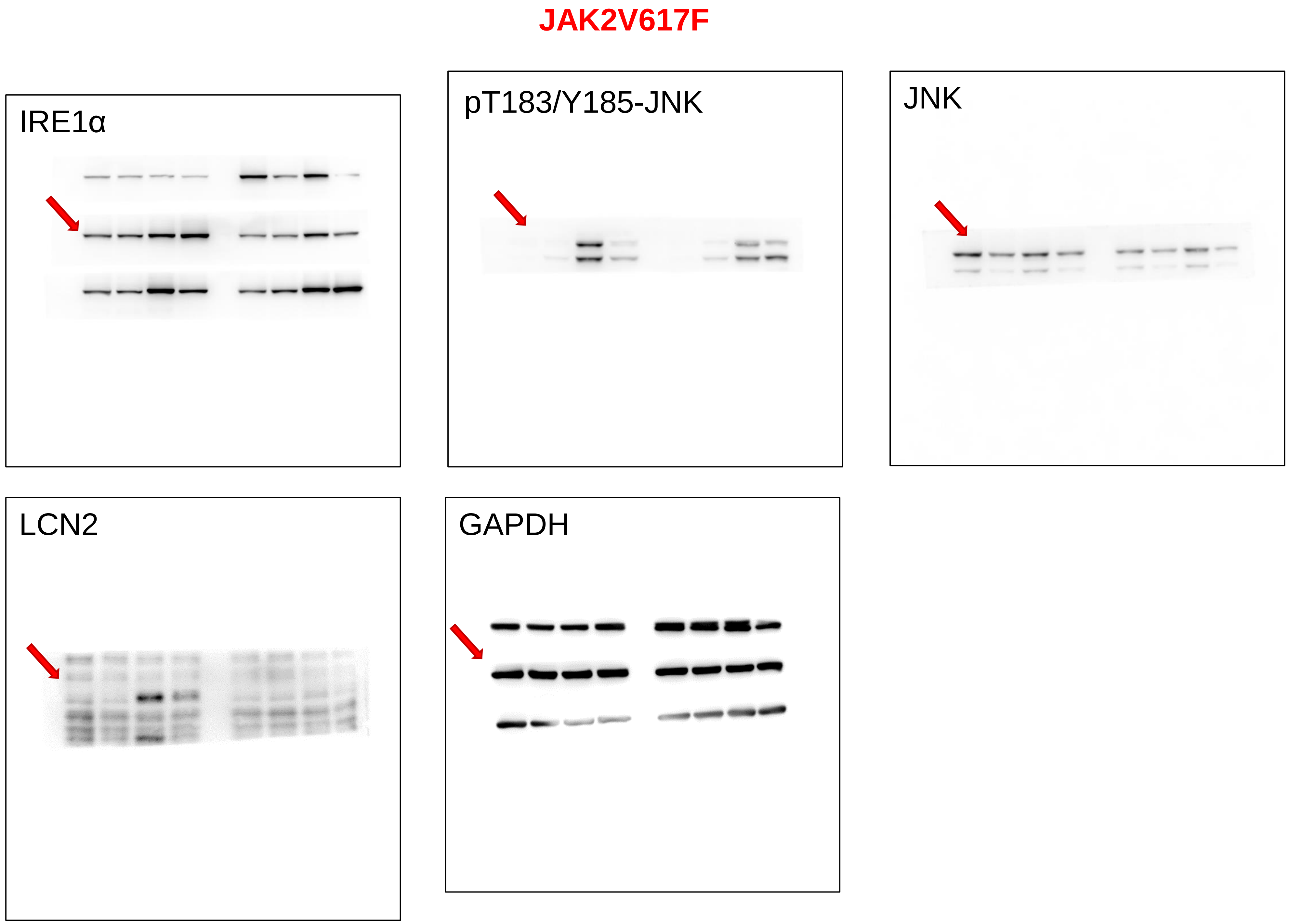

Figure 4C

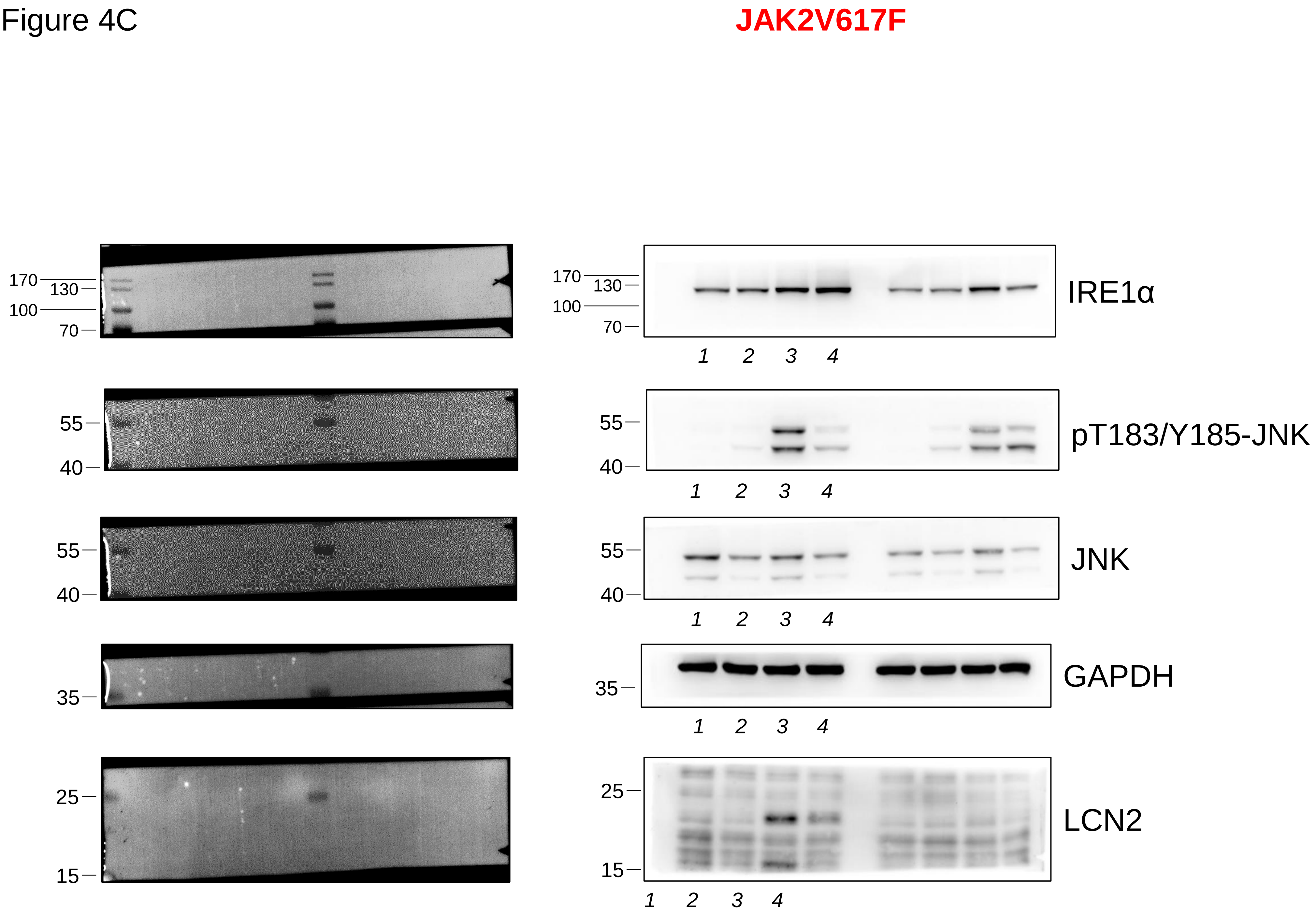

JAK2V617F
170
130
100
70
IRE1α
55
40
pT183/Y185-JNK
JNK
35
GAPDH
25
15
LCN2
1 2 3 4

Figure 4C

JAK2V617F

IRE1α

Lane	optical density
1	3.544.790
2	3.829.447
3	6.727.953
4	7.219.882

pT183/Y185-JNK

Lane	optical density
1	1.305.263
2	2.500.941
3	17.346.966
4	5.053.368

JNK

Lane	optical density
1	6.332.510
2	3.612.104
3	5.569.853
4	3.511.276

GAPDH

Lane	optical density
1	9.967.225
2	9.695.903
3	9.593.196
4	11.090.317

LCN2

Lane	optical density
1	935.770
2	463.749
3	8.634.711
4	4.460.054

Figure 4D

Empty Vector

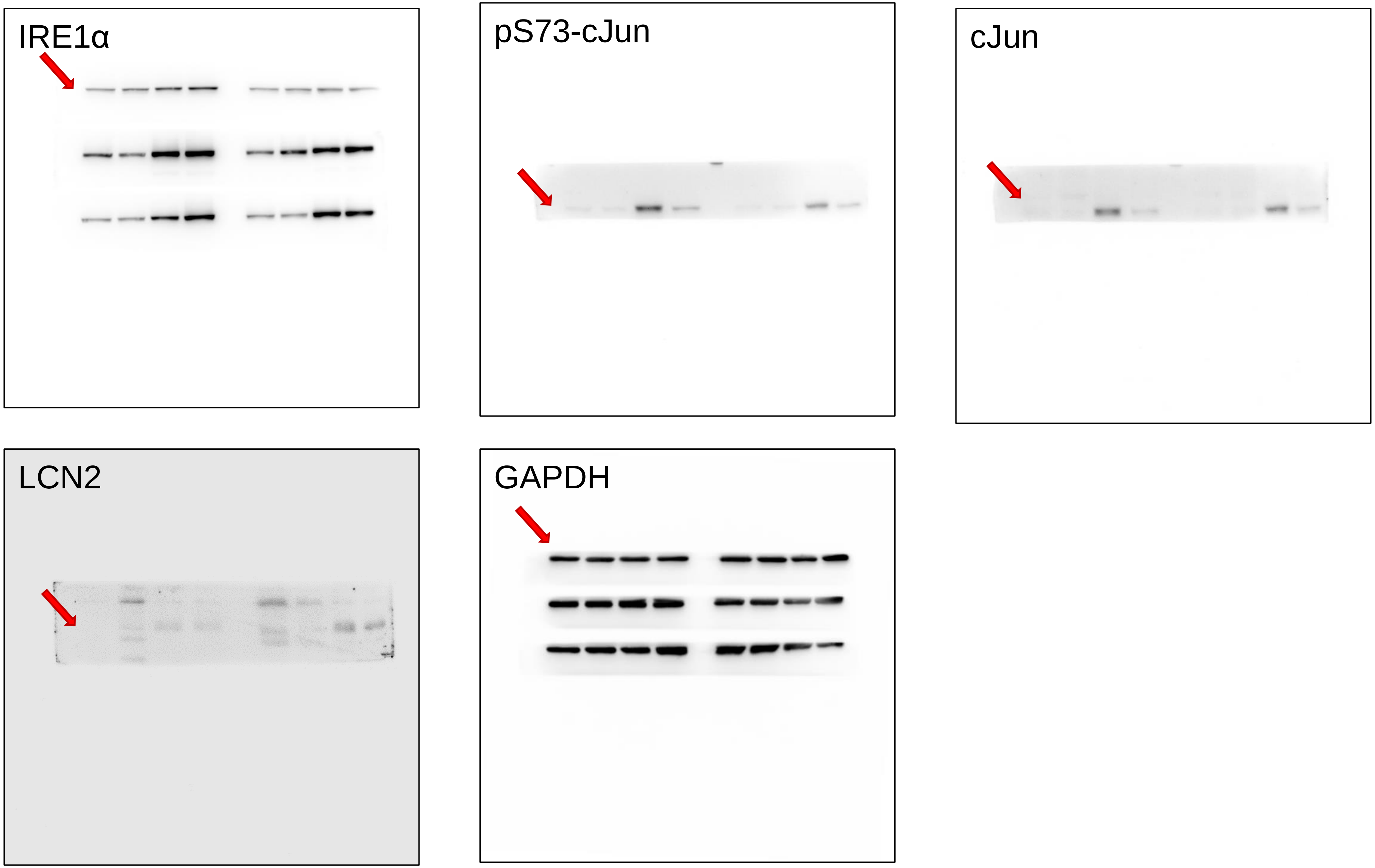

Figure 4D

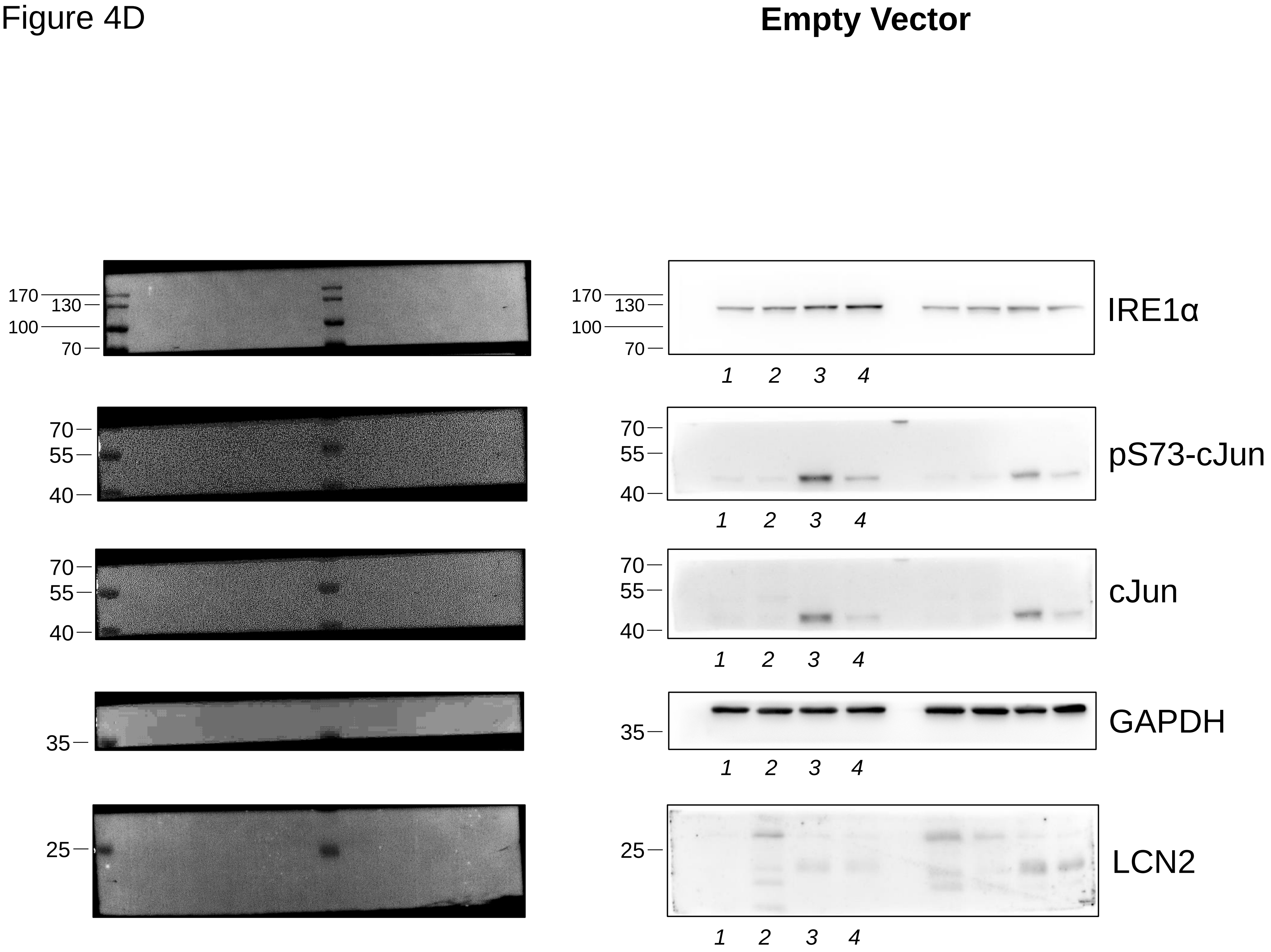

Empty Vector
170
130
100
70
55
40
35
25
1
2
3
4
IRE1α
pS73-cJun
cJun
GAPDH
LCN2

Figure 4D

Empty Vector

IRE1α

Lane	optical density
1	2.004.598
2	2.647.255
3	4.057.154
4	5.191.104

pS73-cJun

Lane	optical density
1	159.607
2	186.021
3	3.435.811
4	873.770

cJun

Lane	optical density
1	95.364
2	74.950
3	2.498.619
4	523.335

GAPDH

Lane	optical density
1	5.873.225
2	5.353.640
3	6.186.589
4	6.525.640

LCN2

Lane	optical density
1	29.950
2	133.364
3	418.849
4	283.192

Figure 4D

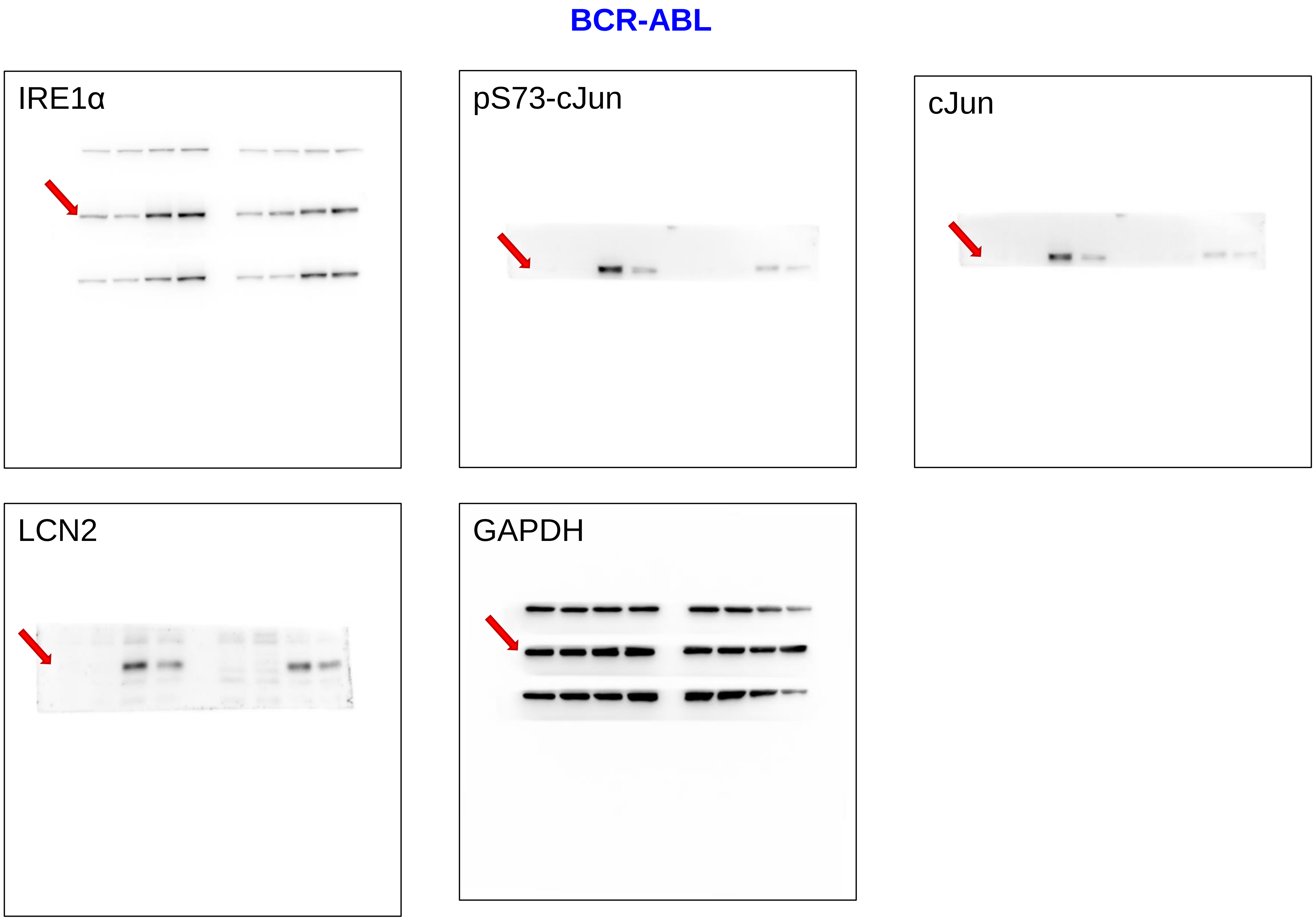

Figure 4D

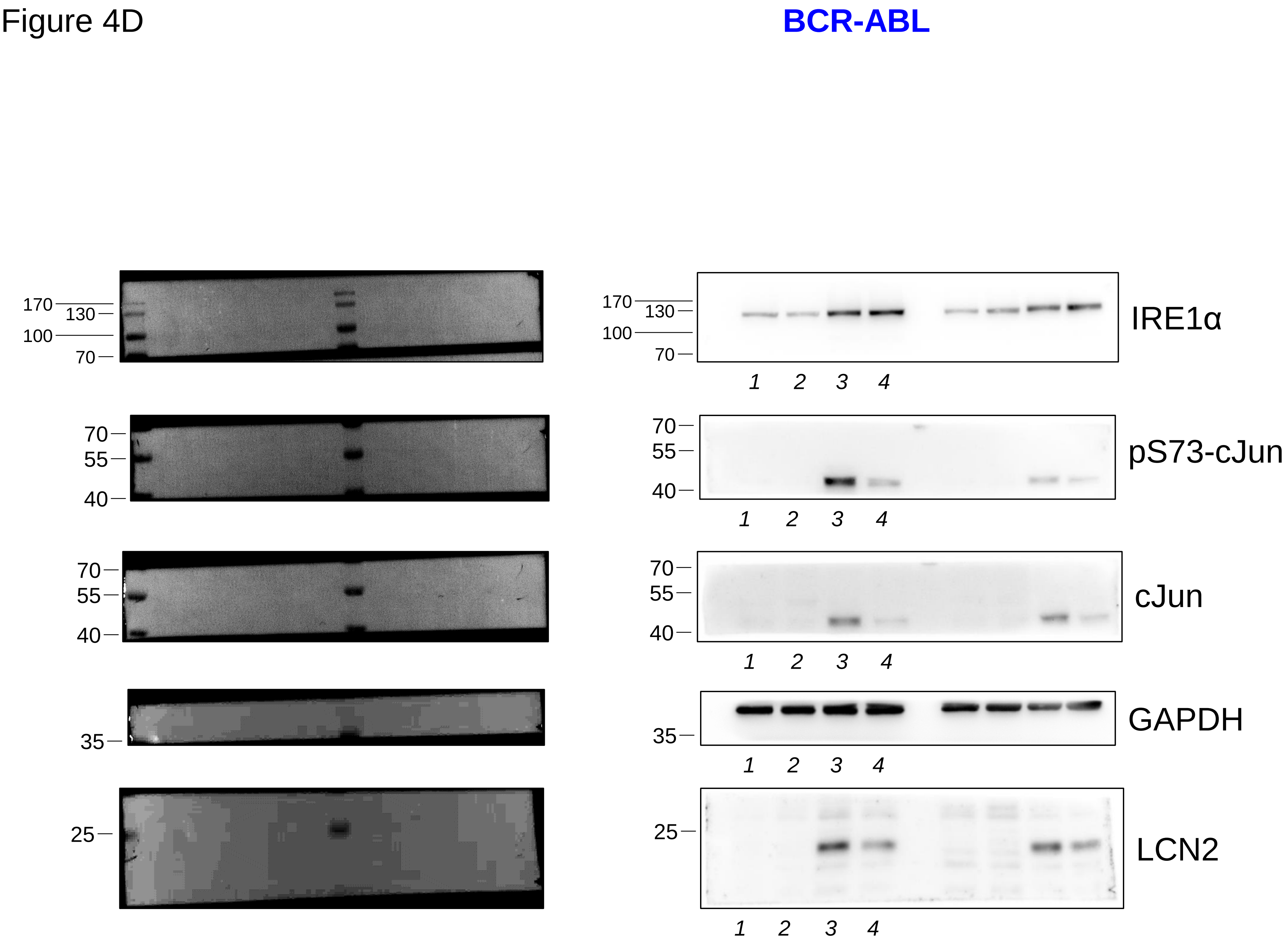
BCR-ABL
170
130
100
70
IRE1α
1 2 3 4
70
55
40
pS73-cJun
1 2 3 4
70
55
40
cJun
1 2 3 4
35
GAPDH
1 2 3 4
25
LCN2
1 2 3 4

Figure 4D

BCR-ABL

IRE1α

Lane	optical density
1	1.326.648
2	1.005.527
3	4.318.104
4	5.622.589

pS73-cJun

Lane	optical density
1	24.657
2	29.364
3	5.671.054
4	1.280.598

cJun

Lane	optical density
1	23.536
2	39.364
3	2.440.154
4	353.678

GAPDH

Lane	optical density
1	8.600.640
2	8.017.518
3	9.132.225
4	10.307.175

LCN2

Lane	optical density
1	80.950
2	34.071
3	10.145.903
4	4.974.175

Figure 4D

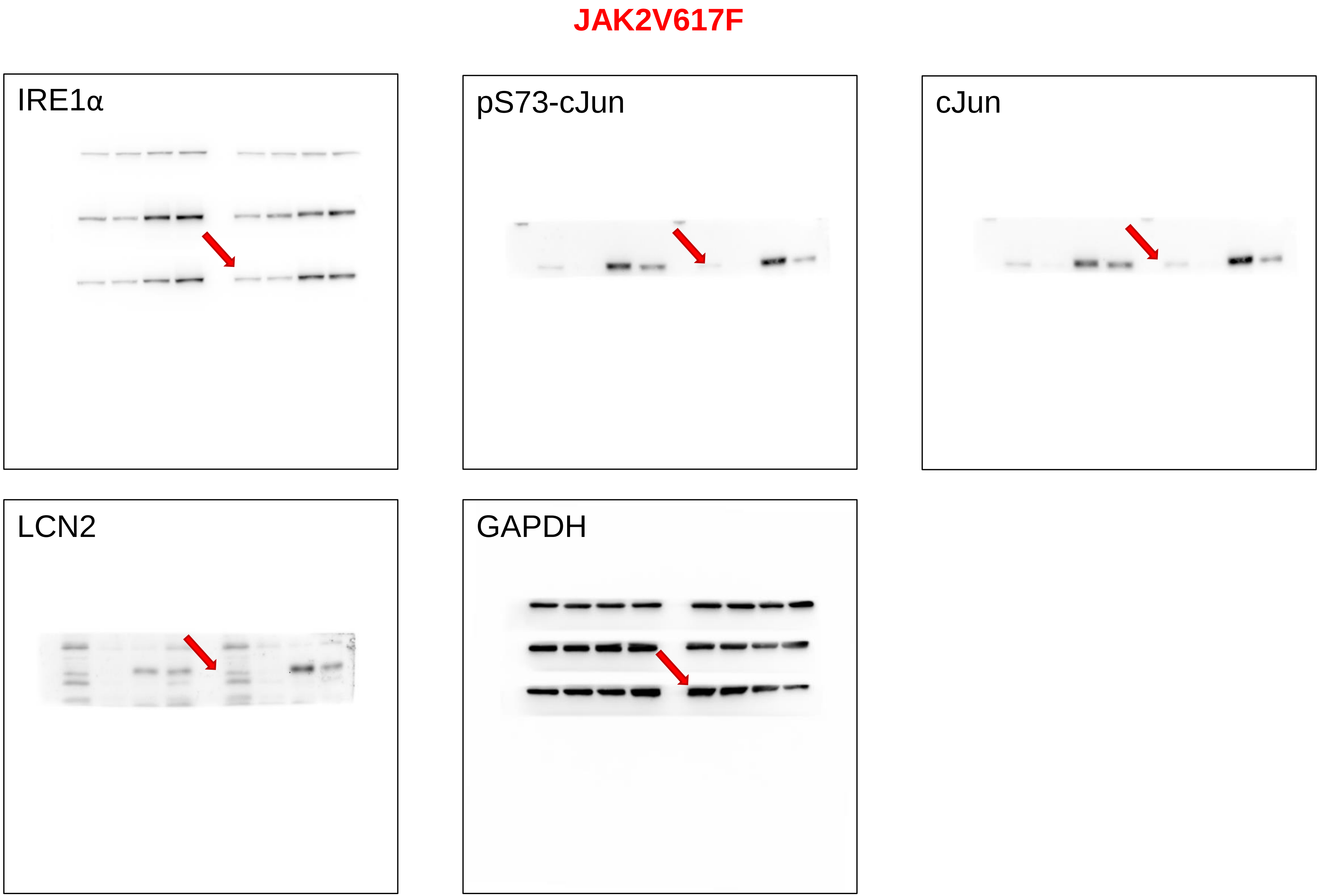

Figure 4D

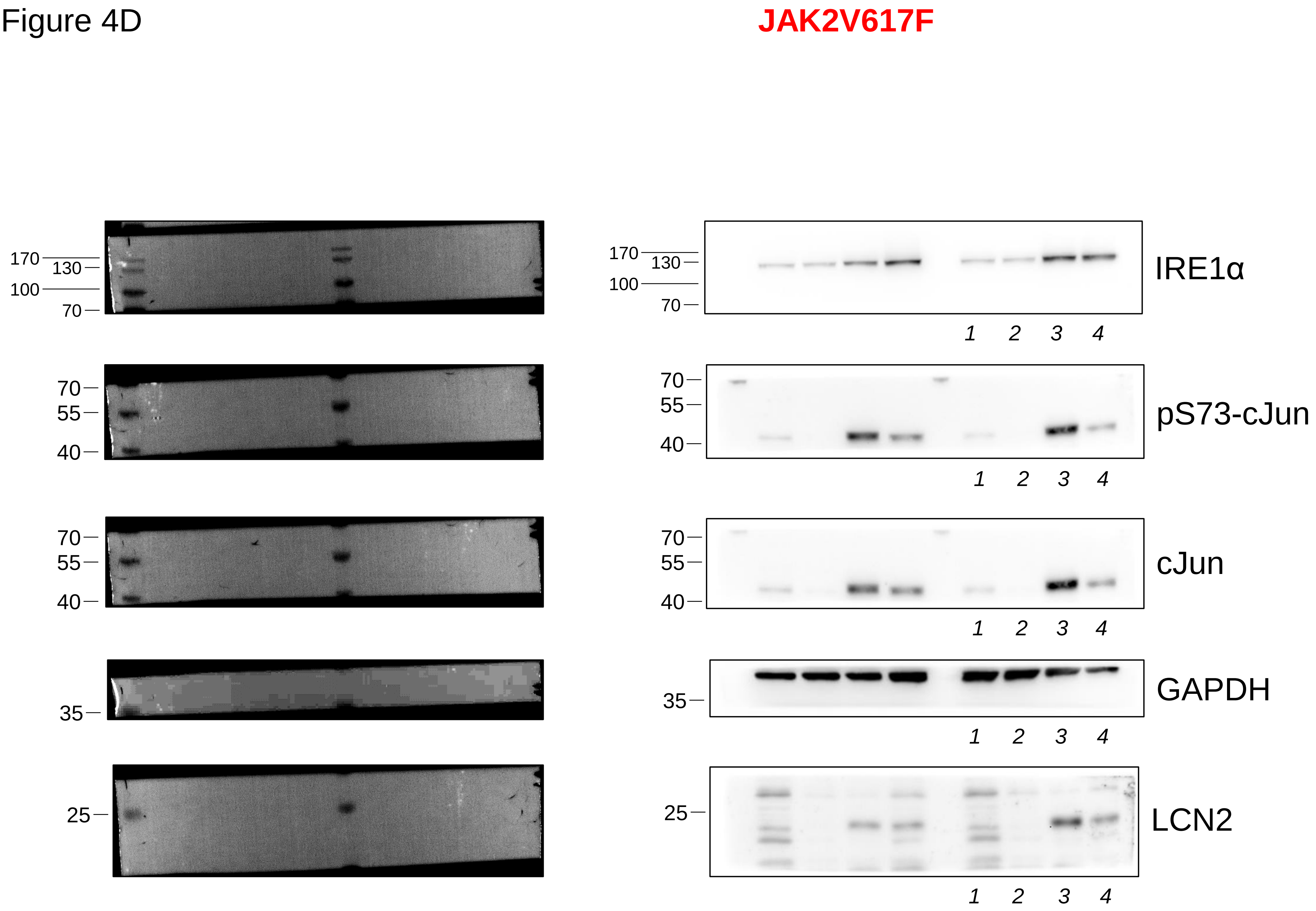
JAK2V617F
170
130
100
70
70
55
40
70
55
40
35
25
170
130
100
70
IRE1α
1 2 3 4
70
55
40
pS73-cJun
1 2 3 4
70
55
40
cJun
1 2 3 4
35
GAPDH
1 2 3 4
25
LCN2
1 2 3 4

Figure 4D

JAK2V617F

IRE1α

Lane	optical density
1	996.648
2	1.062.770
3	4.503.397
4	3.765.740

pS73-cJun

Lane	optical density
1	247.435
2	28.536
3	6.226.589
4	1.189.012

cJun

Lane	optical density
1	309.263
2	55.536
3	6.474.418
4	1.587.497

GAPDH

Lane	optical density
1	10.905.761
2	10.319.468
3	6.896.811
4	5.248.397

LCN2

Lane	optical density
1	701.477
2	127.485
3	9.498.832
4	3.654.276
