# Supplementary material for: The Unfolded Protein Response Is a Major Driver of LCN2 Expression in BCR–ABL- and JAK2V617F-Positive MPN
Source: Cancers (Basel). 2021 Aug 21;13(16):4210. doi: 10.3390/cancers13164210 (PMC8391615; doi:10.3390/cancers13164210)
Supplement: Supplementary file 1 [file cancers-13-04210-s001.zip › cancers-1268224-Suppl.pdf]

cancers

MDPI

# Supplementary Materials: The Unfolded Protein Response Is a Major Driver of LCN2 Expression in BCR–ABL- and JAK2V617F-Positive MPN

Stefan Tillmann, Kathrin Olschok, Sarah K. Schröder, Marlena Bütow, Julian Baumeister, Milena Kalmer, Vera Preußger, Barbora Weinbergerova, Kim Kricheldorf, Jiri Mayer, Blanka Kubesova, Zdenek Racil, Martina Wessiepe, Jörg Eschweiler, Susanne Isfort, Tim H. Brümmendorf, Walter Becker, Mirle Schemionek, Ralf Weiskirchen, Steffen Koschmieder and Nicolas Chatain

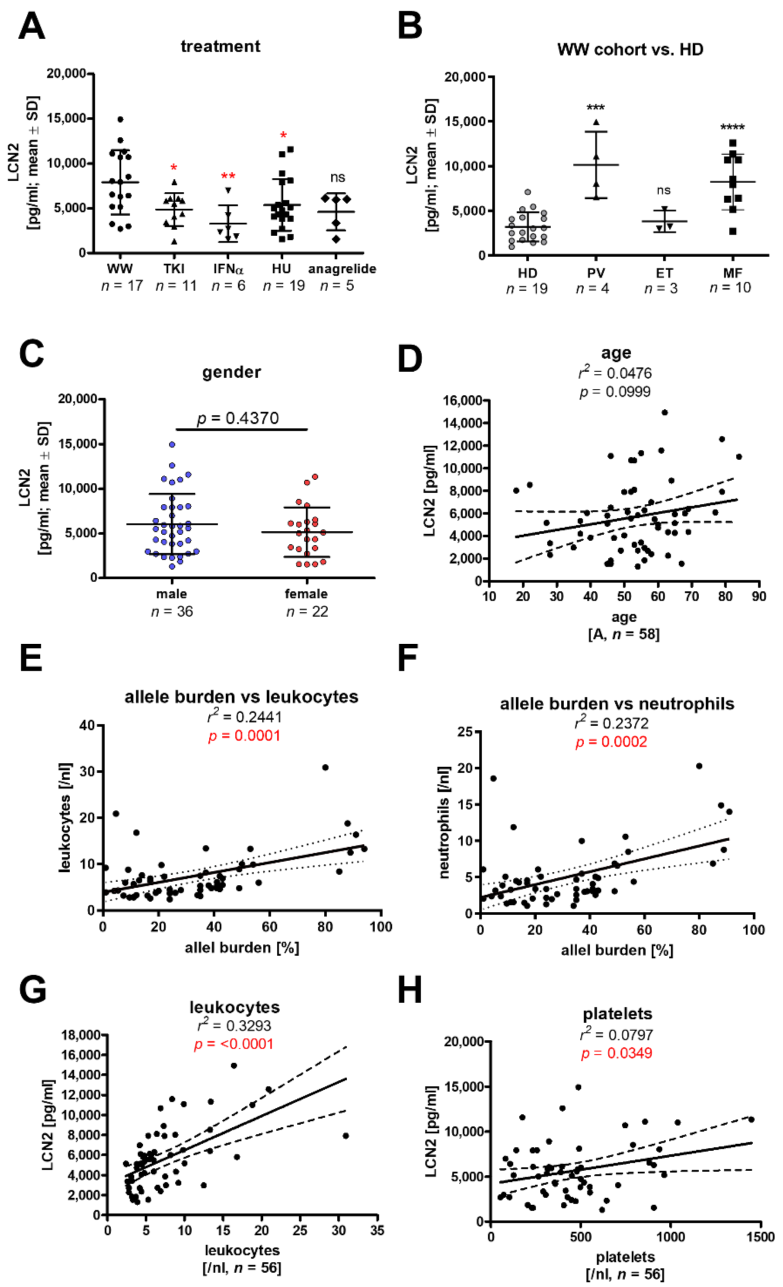


**Figure S1.** Additional information on patient samples used in Figure 2. (**A**) Patient samples sorted by treatment (WW = watch and wait, TKI = JAK2 inhibitor, IFN-$\alpha$ = interferon $\alpha$, HU = hydroxyurea).

Mann-Whitney test. (**B**) Untreated Patient samples (WW cohort) compared to HD. Mann-Whitney test. (**C**) Patient samples from Figure 2 sorted by gender. (**D, G** and **H**) Correlation between LCN2 serum levels and age (**D**), leukocyte count (**G**) or platelets (**H**) in all analyzed patients. Linear regression analysis. (**E** & **F**) correlation between allelic burden and (**E**) leukocytes or (**F**) neutrophils. Linear regression analysis. ns – not significant.

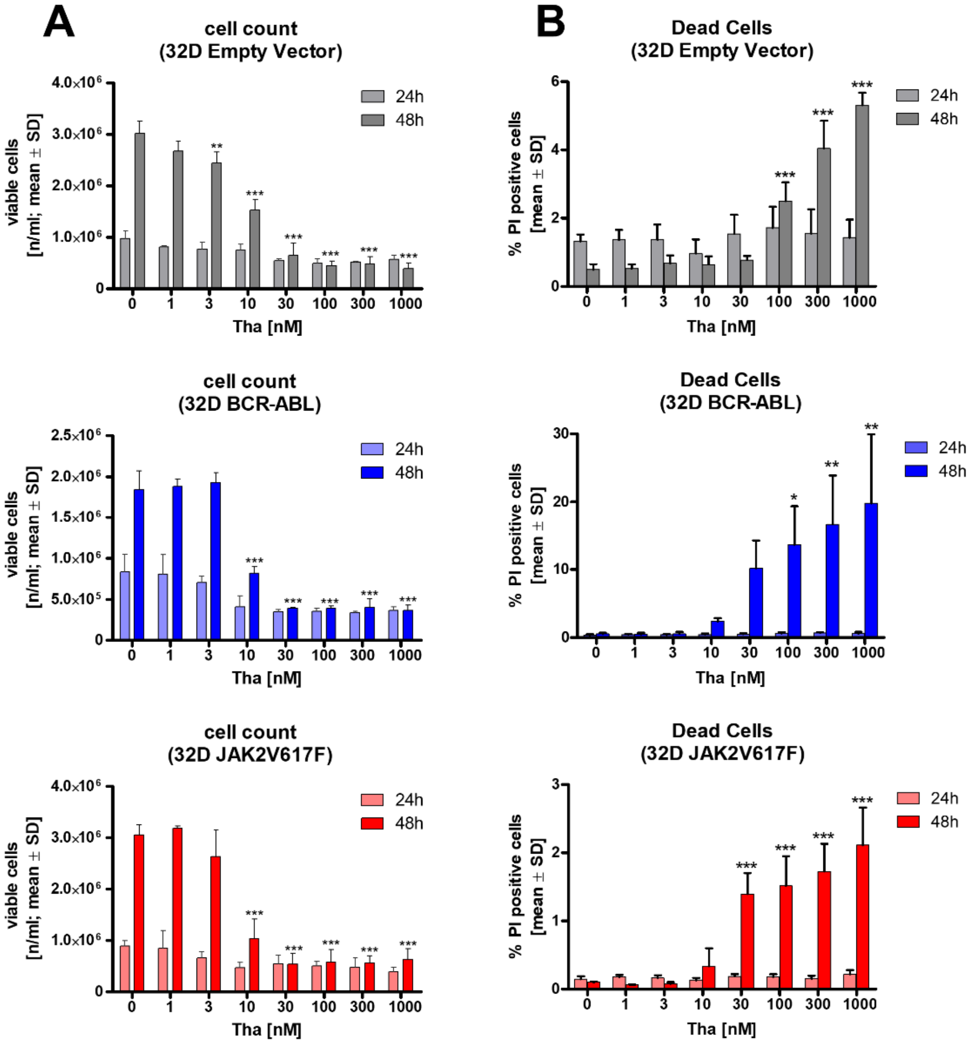


**Figure S2.** Thapsigargin effects growth kinetics of 32D cells. 2 × $10^6$ 32D cells were treated with increasing concentrations of Tha. After 24 h and 48 h, cell numbers were assessed (**A**) and stained for Dead cells using propidium iodide (PI) (**B**). $n = 3$, ANOVA followed by Dunnet's test Asterisks indicate p-values of: * = $p < 0.05$; ** = $p < 0.01$; *** = $p < 0.001$

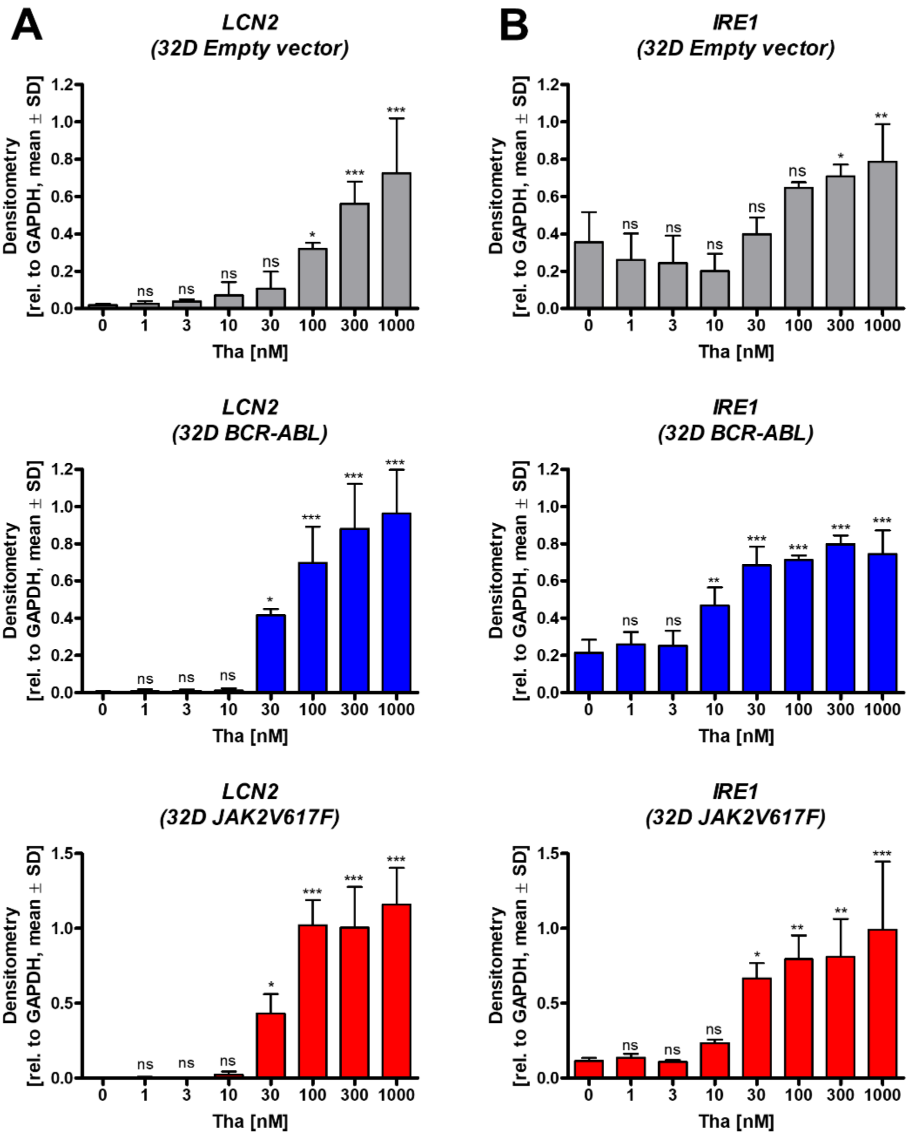


**Figure S3.** Densitometry analysis of LCN2 and IRE1 protein. (**A** & **B**) Densitometry analysis of LCN2 (**A**) and IRE1 (**B**) proteins as determined by Western blot analysis depicted in Figure 3D. $n = 3$, ANOVA followed by Dunnett's test. Asterisks indicate *p*-values of: * = $p < 0.05$; ** = $p < 0.01$; *** = $p < 0.001$. ns – not significant.

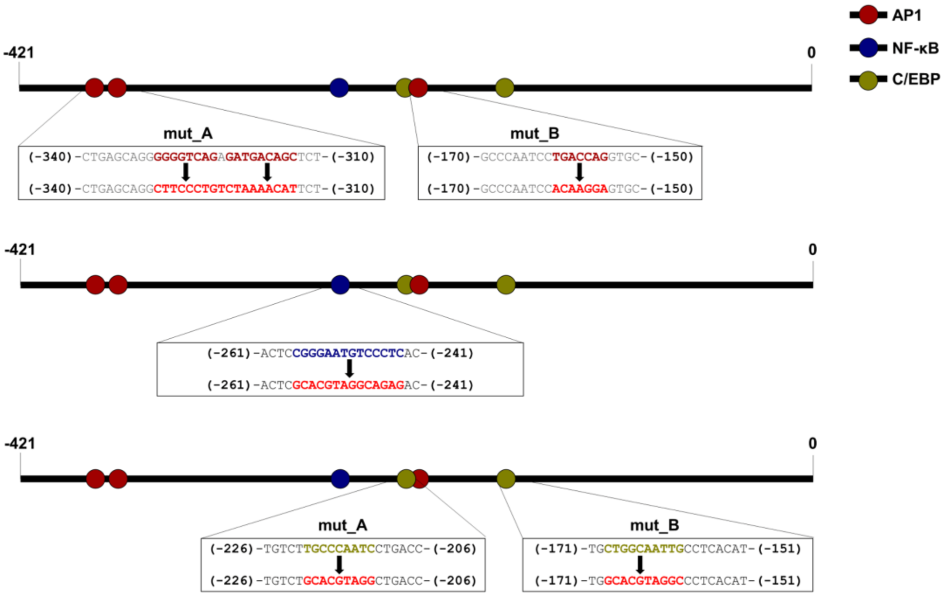


**Figure S4.** Schematic representation of the *LCN2* promoter. Predicted transcription factor binding sites (TFBS) in the 421 bp region upstream of translational start site of the human *LCN2* promoter (0). The boxes show the respective sequences of the TFBS (dark red, blue or yellow), and the changes inserted by site directed mutagenesis (new sequence indicated in red).

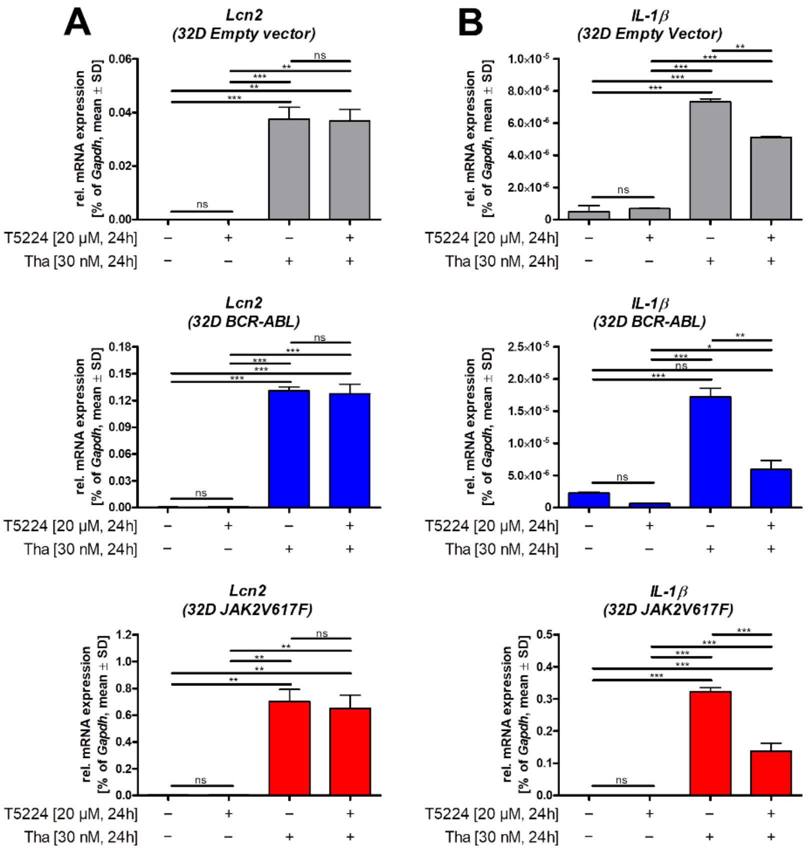


**Figure S5.** Inhibition of AP-1 binding by T5224. (A & B) RT-qPCR analysis of 32D cells treated with 30 nM Tha alone or in combination with 20 µM T5224 for 24 h. $n = 3$, ANOVA followed by Dunnett's test. Asterisks indicate $p$-values of: * = $p < 0.05$; ** = $p < 0.01$; *** = $p < 0.001$. ns – not significant.

**Table S1.** Patient data for experiments using PBMCs and BMMCs.

| Figure | Sample | Mutation | Allele Burden (%) | Diagnosis | Therapy | Leukocytes (/nL) | Monocytes (/nL) | Neutrophils (/nL) | Platelets (/nL) | Hb (g/dL) | Hematocrit (%) | Blasts PB (%) | Blasts BM (%) |
|---|---|---|---|---|---|---|---|---|---|---|---|---|---|
| 1A | PB | *JAK2*V617F | N/A | MF | N/A | N/A | N/A | N/A | N/A | N/A | N/A | N/A | N/A |
| 1A | PB | *JAK2*V617F | N/A | PV | N/A | N/A | N/A | N/A | N/A | N/A | N/A | N/A | N/A |
| 1A | PB | *JAK2*V617F | N/A | MF | JAK2-Inhibitor | 21.4 | 3 | 16.4 | 83 | 10.4 | 36 | N/A | N/A |
| 1A | PB | *JAK2*V617F | N/A | MF | WW | 13.6 | 3 | 11.4 | 473 | 16.9 | 56 | N/A | N/A |
| 1A | PB | *JAK2*V617F | N/A | ET | Hydroxyurea | 5.9 | N/A | N/A | 477 | 14.8 | 44 | N/A | N/A |
| 1A | PB | *JAK2*V617F | N/A | PV | PHL; ASA (WW) | 13.5 | 1 | 12.8 | 628 | 12.9 | 41 | N/A | N/A |
| 1A | PB | *JAK2*V617F | N/A | PV | PHL; ASA (WW) | 7.8 | 8 | 6.0 | 579 | 15.4 | 46 | N/A | N/A |
| 1A | PB | *JAK2*V617F | N/A | ET | Hydroxyurea | 4.4 | 4 | 2.9 | 276 | 14.9 | 24 | N/A | N/A |
| 1A | PB | *JAK2*V617F | N/A | PV | PHL; ASA (WW) | 8.1 | 8 | 5.0 | 393 | 14.7 | 48 | N/A | N/A |
| 1A | PB | *JAK2*V617F | N/A | ET | Anagrelide | 20.5 | 6 | 16.6 | 342 | 11.8 | 38 | N/A | N/A |
| 1A | PB | *JAK2*V617F | N/A | ET | Anagrelide | 6.3 | 8 | 3.5 | 256 | 15 | 44 | N/A | N/A |
| 1A | PB | *JAK2*V617F | N/A | ET | Hydroxyurea | 7.9 | N/A | N/A | 447 | 11 | 33.2 | N/A | N/A |
| 1A | PB | *JAK2*V617F | N/A | MF | Hydroxyurea | 12.4 | N/A | N/A | 157 | 12.5 | 40.5 | N/A | N/A |
| 1A | PB | *JAK2*V617F | N/A | PV | N/A | N/A | N/A | N/A | N/A | N/A | N/A | N/A | N/A |
| 1A | PB | *JAK2*V617F | N/A | PV | N/A | N/A | N/A | N/A | N/A | N/A | N/A | N/A | N/A |
| 1A | PB | *JAK2*V617F | N/A | MF | Hydroxyurea | 7.2 | 5.5 | 4.9 | 412 | 16.5 | 46.9 | N/A | N/A |
| 1A | PB | *BCR-ABL* | N/A | CML | none | N/A | N/A | N/A | N/A | N/A | N/A | N/A | N/A |

| | | | | | | | | | | | | | |
|---|---|---|---|---|---|---|---|---|---|---|---|---|---|
| 1A | PB | *BCR-ABL* | N/A | CML | none | 390 | N/A | N/A | 125 | N/A | N/A | 4% | 2% |
| 1A | PB | *BCR-ABL* | N/A | CML | none | N/A | N/A | N/A | N/A | N/A | N/A | 1% | 2% |
| 1A | PB | *BCR-ABL* | N/A | CML | none | N/A | N/A | N/A | N/A | N/A | N/A | N/A | N/A |
| 1A | PB | *BCR-ABL* | N/A | CML | none | N/A | N/A | N/A | N/A | N/A | N/A | 2% | 4% |
| 4E | PB | *JAK2*V617F | 22 | PV | JAK2-Inhibitor | 7.2 | 5,2 | 4.8 | 443 | 13.1 | 40 | N/A | N/A |
| 4E | PB | *JAK2*V617F | 7 | PV | PHL; ASA (WW) | 5 | 7 | 3.4 | 515 | 13.7 | 43.7 | N/A | N/A |
| 4E | PB | *JAK2*V617F | 17 | PV | Hydroxyurea | 6.7 | 6,1 | 4.4 | 513 | 15.6 | 46.9 | N/A | N/A |
| 1B | BM | *JAK2*V617F | 40.7 | PV | PHL; LMWH | 11.6 | N/A | 8.64 | 649 | 15.8 | 52 | N/A | N/A |
| 1B | BM | *JAK2*V617F | 24.6 | PV | PHL; ASA | 12.3 | N/A | 8.29 | 492 | 14.4 | 43 | N/A | N/A |
| 1B | BM | *JAK2*V617F | 46.9 | PV | ASA | 22.4 | N/A | 17.2 | 544 | 15.0 | 46 | N/A | N/A |
| 1B | BM | *JAK2*V617F | 54.4 | PV | PHL; ASA | 9.71 | N/A | 7.37 | 744 | 15.2 | 46 | N/A | N/A |
| 1B | BM | *JAK2*V617F | 33.3 | PV | ASA | 8.56 | N/A | 5.98 | 684 | 14.5 | 43 | N/A | N/A |
| 1B | BM | *JAK2*V617F | 35.4 | ET | none | 9.28 | N/A | 6.45 | 965 | 16.4 | 50 | N/A | N/A |
| 1B | BM | *JAK2*V617F | 13.6 | ET | none | 8.48 | N/A | 6.01 | 516 | 16.0 | 47 | N/A | N/A |
| 1B | BM | *JAK2*V617F | 50.5 | ET | none | 8.22 | N/A | 5.92 | 486 | 15.1 | 45 | N/A | N/A |
| 1B | BM | *JAK2*V617F | 26.6 | ET | none | 6.11 | N/A | 3.3 | 677 | 14.1 | 42 | N/A | N/A |
| 1B | BM | *JAK2*V617F | 18.5 | ET | none | 10.3 | N/A | 7.75 | 803 | 1.4 | 44 | N/A | N/A |
| 1B | BM | *JAK2*V617F | 91.8 | MF | none | 29.5 | N/A | 28.6 | 609 | 9.6 | 29 | N/A | N/A |
| 1B | BM | *JAK2*V617F | 89.1 | MF | none | 12.3 | N/A | 9.9 | 77 | 11.4 | 34 | N/A | N/A |
| 1B | BM | *JAK2*V617F | 48.4 | MF | none | 29.2 | N/A | 24.6 | 230 | 11.1 | 35 | N/A | N/A |
| 1B | BM | *JAK2*V617F | N/A | MF | none | 46.97 | N/A | 40.63 | 636 | 14.6 | 48 | N/A | N/A |
| 1B | BM | *JAK2*V617F | 2.7 | MF | none | 13.2 | N/A | 8.12 | 734 | 15.2 | 48 | N/A | N/A |
| 1B | BM | *BCR-ABL* | N/A | CML | none | N/A | N/A | N/A | 320 | N/A | N/A | 1% | 2% |

| | | | | | | | | | | | | | |
|---|---|---|---|---|---|---|---|---|---|---|---|---|---|
| 1B | BM | *BCR-ABL* | N/A | CML | none | N/A | N/A | N/A | 782 | N/A | N/A | 0% | 2% |
| 1B | BM | *BCR-ABL* | N/A | CML | none | N/A | N/A | N/A | 287 | N/A | N/A | N/A | N/A |
| 1B | BM | *BCR-ABL* | N/A | CML | none | N/A | N/A | N/A | 125 | N/A | N/A | 4% | 2% |
| 1B | BM | *BCR-ABL* | N/A | CML | none | N/A | N/A | N/A | N/A | N/A | N/A | N/A | N/A |

BM-bone marrow; PB-pheripheral blood; CML-chronic myeloid leukemia; ET-essential thrombocythemia; Hb-hemoglobin; MF-myelofibrosis; PV-polycythemia vera; WW-watch and wait; PHL-phlebotomy; ASA-aspirin; LMWH-low molecular weight heparin; N/A-not available.

**Table S2.** Patient data for experiments using serum.

| Mutation | Allele Burden (%) | Diagnosis | Sex | Age | LCN2 (pg/mL) | Therapy | Leukocytes (/nL) | Monocytes (/nL) | Neutrophils (/nL) | Platelets (/nL) | Hb (g/dL) | Hematocrit (%) |
|---|---|---|---|---|---|---|---|---|---|---|---|---|
| *CALRdel19* | 42 | ET | m | 50 | 7905 | HU | 7.4 | 0.64 | 5.1 | 233 | 14.2 | 42 |
| *CALRdel31* | 42 | preMF | m | 54 | 5845 | IFN | 4.6 | 0.59 | 2.6 | 317 | 13.3 | 39.4 |
| *CALRdel52* | 35 | ET | f | 46 | 1559 | anagrelide | 5.3 | 0.33 | 3.4 | 906 | 11.8 | 35.8 |
| *CALRdel52* | 49 | ET | m | 65 | 5935 | anagrelide | 4.8 | 0.43 | 3.1 | 338 | 14.9 | 45.8 |
| *CALRdel52* | 37 | ET | f | 68 | 6008 | anagrelide | 8.2 | 0.78 | 5.5 | 501 | 10.5 | 29.5 |
| *CALRdel52* | 24 | ET | f | 46 | 1833 | HU | 3.7 | 0.31 | 2.2 | 406 | 10.6 | 33.5 |
| *CALRdel52* | 26 | ET | m | 60 | 4735 | HU | 3.8 | 0.52 | 2.0 | 500 | 13.7 | 39.7 |
| *CALRdel52* | 19 | ET | f | 45 | 5103 | HU | 3.8 | 0.30 | 2.1 | 450 | 11.2 | 34.8 |
| *CALRdel52* | 38 | ET | m | 52 | 5569 | HU | 5.4 | 0.38 | 3.0 | 478 | 15.3 | 41.7 |
| *CALRdel52* | 16 | ET | m | 56 | 1859 | IFN | 3.2 | 0.37 | 1.5 | 205 | 15 | 45.1 |
| *CALRdel52* | 14 | ET | m | 35 | 2998 | WW | 7.6 | 0.93 | 4.3 | 305 | 15.5 | 45.9 |
| *CALRdel52* | 50 | ET | m | 27 | 5180 | WW | 10 | 0.95 | 6.6 | 964 | 11.8 | 37.3 |
| *CaLRdel52* | 53 | MF | f | 22 | 8532 | WW | 13.3 | 0.63 | 10.6 | 790 | 11.9 | 36.5 |
| *CALRdel52* | 41 | preMF | f | 56 | 6285 | WW | 6.1 | 0.50 | 4.1 | 905 | 13.7 | 40.8 |
| *CALRdel52* | 52 | preMF | m | 52 | 10710 | WW | N/A | N/A | N/A | N/A | N/A | N/A |

| | | | | | | | | | | | | |
|---|---|---|---|---|---|---|---|---|---|---|---|---|
| *CALRin5* | 94 | PMF | m | 69 | 6416 | WW | 13.3 | N/A | N/A | 108 | 8.2 | 26.1 |
| *CALRins5* | 17 | ET | f | 28 | 3363 | anagrelide | 2.6 | 0.31 | 1.1 | 291 | 14 | 41.5 |
| *CALRins5* | 43 | MF | f | 51 | 6132 | anagrelide | 5.5 | 0.65 | 3.2 | 396 | 13.2 | 40.8 |
| *CALRins5* | 28 | preMF | m | 50 | 4059 | HU & Jak2 inhibitor | 4.4 | 0.59 | 2.7 | 707 | 15.1 | 42.9 |
| *CALRins5* | 35 | MF | m | 47 | 3833 | HU | 3.1 | 0.25 | 1.9 | 498 | 15.6 | 45.9 |
| *CALRins5* | 35 | MF | m | 40 | 3859 | HU | 7.5 | 1.22 | 4.7 | 550 | 15 | 42.5 |
| *CALRins5* | 34 | ET | f | 67 | 1560 | HU | 3.3 | 0.47 | 1.1 | 228 | 8.7 | 24.9 |
| *CALRins5* | 39 | ET | m | 37 | 4212 | IFN | 4.8 | 0.53 | 3.1 | 359 | 15.1 | 45.6 |
| *CALRins5* | 35 | MF | m | 28 | 2345 | IFN | 4.4 | 0.59 | 2.6 | 644 | 14.9 | 44.9 |
| *JAK2*V617F | 17 | ET | m | 58 | 2400 | anagrelide | 6.9 | 0.58 | 4.6 | 448 | 14.9 | 44.2 |
| *JAK2*V617F | 13 | ET | f | 54 | 2730 | HU | 6.5 | 0.46 | 4.5 | 426 | 12.3 | 38.3 |
| *JAK2*V617F | 17 | ET | f | 53 | 8102 | HU | 6.4 | 0.51 | 4.2 | 490 | 12.2 | 36.6 |
| *JAK2*V617F | 11 | ET | m | 63 | 2278 | HU | 2.8 | 0.31 | 1.6 | 472 | 12.9 | 36.2 |
| *JAK2*V617F | 88 | ET | m | 84 | 11016 | HU | 18.8 | 1.43 | 14.9 | 1039 | 14.1 | 45.9 |
| *JAK2*V617F | 55 | PV | m | 65 | 4286 | HU | N/A | N/A | N/A | N/A | N/A | N/A |
| *JAK2*V617F | 24 | PV | f | 60 | 5023 | HU | 4.5 | 0.33 | 3.4 | 265 | 12 | 36 |
| *JAK2*V617F | 85 | PV | m | 61 | 11583 | HU | 8.4 | 0.41 | 6.9 | 176 | 13.8 | 45.3 |
| *JAK2*V617F | 49 | PV | f | 63 | 4348 | HU | 8.9 | 0.36 | 6.9 | 355 | 15.9 | 46.5 |
| *JAK2*V617F | 41 | PV | f | 37 | 5340 | HU | 4.8 | 0.40 | 3.1 | 312 | 13.9 | 38.2 |
| *JAK2*V617F | 22 | PV | m | 64 | 8909 | HU | 7.3 | 0.65 | 5.1 | 324 | 14.4 | 42.9 |
| *JAK2*V617F | 12 | ET | f | 45 | 1543 | IFN | 3.3 | 0.35 | 1.6 | 235 | 13.5 | 40.7 |
| *JAK2*V617F | 20 | ET | m | 58 | 6988 | IFN | 4.2 | 0.28 | 3.4 | 82 | 13.7 | 40.7 |
| *JAK2*V617F | 9.60 | PV | m | 49 | 2731 | IFN | 2.8 | 0.29 | 1.4 | 104 | 15.1 | 43.2 |

| | | | | | | | | | | | | |
|---|---|---|---|---|---|---|---|---|---|---|---|---|
| *JAK2*V617F | 89 | MF | m | 56 | 2972 | Jak2 inhibitor | 12.5 | 1.20 | 8.8 | 72 | 13.4 | 39.1 |
| *JAK2*V617F | 12 | MF | m | 45 | 5817 | Jak2 inhibitor | 16.8 | 1.41 | 11.9 | 484 | 14.9 | 43.1 |
| *JAK2*V617F | 5.40 | Post-PV-MF | m | 77 | 6094 | Jak2 inhibitor | 4.3 | 0.31 | 3.2 | 235 | 12.5 | 38.5 |
| *JAK2*V617F | 1.1 | PV | m | 54 | 1311 | Jak2 inhibitor | 3.9 | 0.44 | 2.1 | 619 | 12.5 | 36.7 |
| *JAK2*V617F | 7.40 | PV | f | 55 | 3434 | Jak2 inhibitor | 3.2 | 0.35 | 2.4 | 416 | 12.9 | 39.2 |
| *JAK2*V617F | 56 | PV | f | 69 | 4363 | Jak2 inhibitor | 6 | 0.45 | 4.4 | 518 | 11.3 | 34.2 |
| *JAK2*V617F | 8 | PV | m | 59 | 5494 | Jak2 inhibitor | 6 | 0.51 | 3.9 | 389 | 14.1 | 42 |
| *JAK2*V617F | 11 | PV | f | 39 | 6053 | Jak2 inhibitor | 5.7 | 0.56 | 3.3 | 324 | 11.4 | 34.6 |
| *JAK2*V617F | 80 | PV | m | 79 | 7926 | Jak2 inhibitor | 30.9 | 3.46 | 20.3 | 142 | 9.6 | 35.9 |
| *JAK2*V617F | 0.99 | ET | f | 53 | 3235 | WW | 9.2 | 0.60 | 6.1 | 551 | 14.8 | 45.9 |
| *JAK2*V617F | 4 | MF | m | 57 | 2703 | WW | 4.2 | 0.84 | 2.4 | 54 | 10.8 | 35.6 |
| *JAK2*V617F | 24 | MF | m | 65 | 5156 | WW | 2.4 | 0.31 | 1.3 | 127 | 8.5 | 26.1 |
| *JAK2*V617F | 41 | MF | m | 52 | 7920 | WW | 5.3 | 0.51 | 3.4 | 257 | 14.9 | 45.4 |
| *JAK2*V617F | 43 | MF | f | 53 | 10684 | WW | 6.9 | 0.62 | 4.9 | 746 | 11.2 | 35.3 |
| *JAK2*V617F | 4.70 | MF | m | 79 | 12585 | WW | 20.9 | 1.25 | 18.6 | 399 | 6.9 | 23.3 |
| *JAK2*V617F | 37 | preMF | f | 55 | 11336 | WW | 13.4 | 0.56 | 10.0 | 1448 | 14.9 | 48.3 |
| *JAK2*V617F | 24 | PV | m | 18 | 8037 | PHL; ASA (WW) | 8.8 | 0.54 | 5.1 | 937 | 13.6 | 47 |
| *JAK2*V617F | 54 | PV | m | 46 | 11091 | PHL; ASA (WW) | 9.9 | 0.40 | 8.5 | 857 | 13.5 | 46.9 |
| *JAK2*V617F | 21 | PV | f | 46 | 6519 | PHL; ASA (WW) | 9.8 | 0.45 | 6.1 | 881 | 14.2 | 44.2 |
| *JAK2*V617F | 91 | PV | m | 62 | 14933 | PHL; ASA (WW) | 16.4 | 0.36 | 14.0 | 487 | 12.9 | 45.9 |

ET-essential thrombocythemia; Hb-hemoglobin; HU-hydroxyurea; IFN-interferon alpha; MF-myelofibrosis; PV-polycythemia vera; WW-watch and wait; PHL-phlebotomy; ASA-aspirin; N/A-not available.

**Table S3.** RT-qPCR primers.

| Target | Species | Sequence |
|---|---|---|
| GAPDH_for | Human | 5′-GAAGGTGAAGGTCGGAGT-3′ |
| GAPDH_rev | Human | 5′-GAAGATGGTGATGGGATTTC-3′ |
| LCN2_for | Human | 5′-CTCCACCTCAGACCTGATCC-3′ |
| LCN2_rev | Human | 5′-ACATACCACTTCCCCTGGAAT-3′ |
| MPO_for | Human | 5′-CCGGGATGGTGATCGGTTTT-3′ |
| MPO_rev | Human | 5′-CAGATGATCCGGGGCAATGA-3′ |
| MPL_for | Human | 5′-CTGCCACTTCAAGTCACGAA-3′ |
| MPL_rev | Human | 5′-CTGCCACTCCAATTCCAGAT-3′ |
| EPOR_for | Human | 5′-GAGCATGCCCAGGATACCTA-3′ |
| EPOR_rev | Human | 5′-TACTCAAAGCTGGCAGCAGA-3′ |
| Lcn2_for | Murine | 5′-GCAGGTGGTACGTTGTGGG-3′ |
| Lcn2_rev | Murine | 5′-CTCTTGTAGCTCATAGATGGTGC-3′ |
| Gapdh_for | Murine | 5′-TTGTGCAGTGCCAGCCT-3′ |
| Gapdh_rev | Murine | 5′-CCAATACGGCCAAATCCG-3′ |
| Il-1b_for | Murine | 5′-CAGCTCATATGGGTCCGACA-3′ |
| Il-1b_rev | Murine | 5′-CTGTGTCTTTCCCGTGGACC-3′ |

**Table S4.** Western blot antibodies.

| Target | Supplier | Order No. |
|---|---|---|
| LCN2 | R&D Systems | AF1757 |
| GAPDH | Santa Cruz | sc-32233 |
| IRE1$\alpha$ | Cell Signaling | #3294 |
| CRKL | Cell Signaling | #3182 |
| pT207-CRKL | Cell Signaling | #3181 |
| STAT5 | Santa Cruz | sc-835 |
| pY694-STAT5 | Cell Signaling | #9351 |
| JNK | Cell Signaling | #9252 |
| pT183/Y185-JNK | Cell Signaling | #4668 |
| cJun | Cell Signaling | #9165 |
| pS73-cJun | Cell Signaling | #3270 |

**Table S5.** Cloning primers.

| Target | Species | Sequence |
|---|---|---|
| Lcn2_promotor_1076 _for | Murine | 5′-CATCGGTACCTCTGCCCAAAGTAACTGGAG-3′ |

| | | |
|---|---|---|
| Lcn2_promotor_793_for | Murine | 5′-CATCGGTACCCCACATCTAAGGACTACGTG-3′ |
| Lcn2_promotor_412_for | Murine | 5′-CATCGGTACCAAGCCTGTACTATACTCACT-3′ |
| Lcn2_promotor_189_for | Murine | 5′-CATCGGTACCGAAATCTTGCCAAGTATTTC-3′ |
| Lcn2_promotor_132_for | Murine | 5′-CATCGGTACCGACTTGGTAAAGGATGGACT-3′ |
| Lcn2_promotor_rev | Murine | 5′-CATCAAGCTTTTTCCACAGCTACTAGGTCT-3′ |
| LCN2_promotor_1076_for | Human | 5′-ACTGGTACCTCTCTGCGGACACTGCACAG-3′ |
| LCN2_promotor_793_for | Human | 5′-TCACGGGTACCACACATCAGCCCAACTCAC-3′ |
| LCN2_promotor_412_for | Human | 5′-GGGAGGGTACCACAGGGCACATACCCC-3′ |
| LCN2_promotor_189_for | Human | 5′-TCAGGGTACCAATCTTGCCAAGTGTTTCC-3′ |
| LCN2_promotor_132_for | Human | 5′-TCAGGGTACCTGGCCCGGGCAAAGAATGAATCAACCC-3′ |
| LCN2_promotor_rev | Human | 5′-AGGGCCATGGTTTCAGGGCCGAGGAAGCAG-3′ |
| LCN2_promotor_rev | Human | 5′-CATCAAGCTTTTTCCACAGCTACTAGGTCT-3′ |
| AP1_mut A_for | Human | 5′-CTAAAACATTCTTCCGGCTCACAGGCC-3′ |
| AP1_mut A_rev | Human | 5′-ACAGGGAAGCCTGCTCAGCTGGATCCC-3′ |
| AP1_mut B_for | Human | 5′-GGAGTGCAGAAATCTTGCCAAG-3′ |
| AP1_mut B_rev | Human | 5′-TTGTGGATTGGGCAAGACAGAG-3′ |
| NFκB_mut_for | Human | 5′-GGCAGAGACTCTCCCCGTCCCTCTG-3′ |
| NFκB_mut_rev | Human | 5′-TACGTGCGAGTGCTGCACCTCTGGC-3′ |
| CEBP_mut A_for | Human | 5′-TAGGCTGACCAGGTGCAGAAATCTTG-3′ |
| CEBP_mut A_rev | Human | 5′-CGTGCAGACAGAGGGACGGGGAG-3′ |
| CEBP_mut B_for | Human | 5′-TAGGCCCTCACATTCCTGGCCTT-3′ |
| CEBP_mut B_rev | Human | 5′-CGTGCCAACTCCTGCGGAAACAC-3′ |
